# Effectiveness and cost-effectiveness of four different strategies for SARS-CoV-2 surveillance in the general population (CoV-Surv Study): a structured summary of a study protocol for a cluster-randomised, two-factorial controlled trial

**DOI:** 10.1186/s13063-020-04982-z

**Published:** 2021-01-08

**Authors:** Andreas Deckert, Simon Anders, Manuela de Allegri, Hoa Thi Nguyen, Aurélia Souares, Shannon McMahon, Kathleen Boerner, Matthias Meurer, Konrad Herbst, Matthias Sand, Lisa Koeppel, Tobias Siems, Lucia Brugnara, Stephan Brenner, Robin Burk, Dan Lou, Daniel Kirrmaier, Yuanqiang Duan, Svetlana Ovchinnikova, Michael Marx, Hans Georg Kräusslich, Michael Knop, Till Bärnighausen, Claudia Denkinger

**Affiliations:** 1 Heidelberg Institute of Global Health, University of Heidelberg, Im Neuenheimer Feld 324, 69120 Heidelberg, Germany; 2 Center for Molecular Biology Heidelberg (ZMBH), University of Heidelberg, Im Neuenheimer Feld 282, 69120 Heidelberg, Germany; 3 Heidelberg Institute of Global Health, University of Heidelberg, Im Neuenheimer Feld 130.1, 69120 Heidelberg, Germany; 4 Heidelberg Institute of Global Health, University of Heidelberg, Im Neuenheimer Feld 365, 69120 Heidelberg, Germany; 5 Department of Infectious Diseases, Virology, University of Heidelberg, Im Neuenheimer Feld 267, 69120 Heidelberg, Germany; 6 GESIS Leibniz-Institute for the Social Sciences, B2/1, 68159 Mannheim, Germany; 7 Section Clinical Tropical Medicine, University of Heidelberg, Im Neuenheimer Feld 324, 69120 Heidelberg, Germany; 8 Institute for Applied Mathematics, University of Heidelberg, Berliner Str. 41-49, 69120 Heidelberg, Germany; 9 evaplan GmbH, University of Heidelberg, Ringstr.19b, 69115 Heidelberg, Germany; 10 Heidelberg Institute of Global Health/evaplan GmbH, University of Heidelberg, Im Neuenheimer Feld 130.1, 69120 Heidelberg, Germany; 11 Center for Integrative Infectious Disease Research (CIID), University of Heidelberg, Im Neuenheimer Feld 344, 69120 Heidelberg, Germany

**Keywords:** COVID-19, cluster-randomised controlled trial, protocol, population-based surveillance, cost-effectiveness, implementation, pandemic

## Abstract

**Objectives:**

In this cluster-randomised controlled study (CoV-Surv Study), four different “active” SARS-CoV-2 testing strategies for general population surveillance are evaluated for their effectiveness in determining and predicting the prevalence of SARS-CoV-2 infections in a given population. In addition, the costs and cost-effectiveness of the four surveillance strategies will be assessed. Further, this trial is supplemented by a qualitative component to determine the acceptability of each strategy. Findings will inform the choice of the most effective, acceptable and affordable strategy for SARS-CoV-2 surveillance, with the most effective and cost-effective strategy becoming part of the local public health department’s current routine health surveillance activities. Investigating its everyday performance will allow us to examine the strategy’s applicability to real time prevalence prediction and the usefulness of the resulting information for local policy makers to implement countermeasures that effectively prevent future nationwide lockdowns.

The authors would like to emphasize the importance and relevance of this study and its expected findings in the context of population-based disease surveillance, especially in respect to the current SARS-CoV-2 pandemic. In Germany, but also in many other countries, COVID-19 surveillance has so far largely relied on passive surveillance strategies that identify individuals with clinical symptoms, monitor those cases who then tested positive for the virus, followed by tracing of individuals in close contact to those positive cases.

To achieve higher effectiveness in population surveillance and to reliably predict the course of an outbreak, screening and monitoring of infected individuals without major symptoms (about 40% of the population) will be necessary. While current testing capacities are also used to identify such asymptomatic cases, this rather passive approach is not suitable in generating reliable population-based estimates of the prevalence of asymptomatic carriers to allow any dependable predictions on the course of the pandemic.

To better control and manage the SARS-CoV-2 pandemic, current strategies therefore need to be complemented by an active surveillance of the wider population, i.e. routinely conducted testing and monitoring activities to identify and isolate infected individuals regardless of their clinical symptoms. Such active surveillance strategies will enable more effective prevention of the spread of the virus as they can generate more precise population-based parameters during a pandemic. This essential information will be required in order to determine the best strategic and targeted short-term countermeasures to limit infection spread locally.

**Trial design:**

This trial implements a cluster-randomised, two-factorial controlled, prospective, interventional, single-blinded design with four study arms, each representing a different SARS-CoV-2 testing and surveillance strategy.

**Participants:**

Eligible are individuals age 7 years or older living in Germany’s Rhein-Neckar Region who consent to provide a saliva sample (all four arms) after completion of a brief questionnaire (two arms only). For the qualitative component, different samples of study participants and non-participants (i.e. eligible for study, but refuse to participate) will be identified for additional interviews. For these interviews, only individuals age 18 years or older are eligible.

**Intervention and comparator:**

Of the four surveillance strategies to be assessed and compared, Strategy A1 is considered the gold standard for prevalence estimation and used to determine bias in other arms. To determine the cost-effectiveness, each strategy is compared to status quo, defined as the currently practiced passive surveillance approach.

Strategy A1: Individuals (one per household) receive information and study material by mail with instructions on how to produce a saliva sample and how to return the sample by mail. Once received by the laboratory, the sample is tested for SARS-CoV-2 using *Reverse Transcription Loop-mediated Isothermal Amplification* (RT-LAMP).

Strategy A2: Individuals (one per household) receive information and study material by mail with instructions on how to produce their own as well as saliva samples from each household member and how to return these samples by mail. Once received by the laboratory, the samples are tested for SARS-CoV-2 using RT-LAMP.

Strategy B1: Individuals (one per household) receive information by mail on how to complete a brief pre-screening questionnaire which asks about COVID-19 related clinical symptoms and risk exposures. Only individuals whose pre-screening score crosses a defined threshold, will then receive additional study material by mail with instructions on how to produce a saliva sample and how to return the sample by mail. Once received by the laboratory, the saliva sample is tested for SARS-CoV-2 using RT-LAMP.

Strategy B2: Individuals (one per household) receive information by mail on how to complete a brief pre-screening questionnaire which asks about COVID-19 related clinical symptoms. Only individuals whose pre-screening score crosses a defined threshold, will then receive additional study material by mail with instructions how to produce their own as well as saliva samples from each household member and how to return these samples by mail. Once received by the laboratory, the samples are tested for SARS-CoV-2 using RT-LAMP.

In each strategy, RT-LAMP positive samples are additionally analyzed with qPCR in order to minimize the number of false positives.

**Main outcomes:**

The identification of the one best strategy will be determined by a set of parameters. Primary outcomes include costs per correctly screened person, costs per positive case, positive detection rate, and precision of positive detection rate. Secondary outcomes include participation rate, costs per asymptomatic case, prevalence estimates, number of asymptomatic cases per study arm, ratio of symptomatic to asymptomatic cases per study arm, participant satisfaction.

Additional study components (not part of the trial) include cost effectiveness of each of the four surveillance strategies compared to passive monitoring (i.e. status quo), development of a prognostic model to predict hospital utilization caused by SARS-CoV-2, time from test shipment to test application and time from test shipment to test result, and perception and preferences of the persons to be tested with regard to test strategies.

**Randomisation:**

Samples are drawn in three batches of three continuous weeks. Randomisation follows a two-stage process. First, a total of 220 sampling points have been allocated to the three different batches. To obtain an integer solution, the Cox-algorithm for controlled rounding has been used. Afterwards, sample points have been drawn separately per batch, following a probability proportional to size (PPS) random sample. Second, for each cluster the same number of residential addresses is randomly sampled from the municipal registries (self-weighted sample of individuals). The 28,125 addresses drawn per municipality are then randomly allocated to the four study arms A1, A2, B1, and B2 in the ratio 5 to 2.5 to 14 to 7 based on the expected response rates in each arm and the sensitivity and specificity of the pre-screening tool as applied in strategy B1 and B2. Based on the assumptions, this allocation should yield 2500 saliva samples in each strategy. Although a municipality can be sampled by multiple batches and the overall number of addresses per municipality might vary, the number of addresses contacted in each arm is kept constant.

**Blinding (masking):**

The design is single-blinded, meaning the staff conducting the SARS-CoV-2 tests are unaware of the study arm assignment of each single participant and test sample.

**Sample sizes:**

Total sample size for the trial is 10,000 saliva samples equally allocated to the four study arms (i.e. 2,500 participants per arm).

For the qualitative component, up to 60 in-depth interviews will be conducted with about 30 study participants (up to 15 in each arm A and B) and 30 participation refusers (up to 15 in each arm A and B) purposefully selected from the quantitative study sample to represent a variety of gender and ages to explore experiences with admission or rejection of study participation. Up to 25 asymptomatic SARS-CoV-2 positive study participants will be purposefully selected to explore the way in which asymptomatic men and women diagnosed with SARS-CoV-2 give meaning to their diagnosis and to the dialectic between feeling concurrently healthy and yet also being at risk for transmitting COVID-19. In addition, 100 randomly selected study participants will be included to explore participants’ perspective on testing processes and implementation.

**Trial Status:**

Final protocol version is “Surveillance_Studienprotokoll_03Nov2020_v1_2” from November 3, 2020. Recruitment started November 18, 2020 and is expected to end by or before December 31, 2020.

**Trial registration:**

The trial is currently being registered with the German Clinical Trials Register (Deutsches Register Klinischer Studien), DRKS00023271 (https://www.drks.de/drks_web/navigate.do?navigationId=trial. HTML&TRIAL_ID=DRKS00023271). Retrospectively registered 30 November 2020.

**Full protocol:**

The full protocol is attached as an additional file, accessible from the Trials website (Additional file 1). In the interest in expediting dissemination of this material, the familiar formatting has been eliminated; this Letter serves as a summary of the key elements of the full protocol.

## Supplementary Information

**Additional file 1.**

## Data Availability

The quantitative data will populate the National COVID-19 Research Network (Nationales COVID-19 Forschungsnetzwerk) and become available in an anonymized form to other researchers. The goal of this research network is to combine all SARS-CoV-2-specific data in Germany to allow for additional joint analyses. This includes plans for the establishment of an overarching data structure based on standardized variable definitions. Researchers outside the network will be asked to follow an application process prior to data access. On a case-to-case basis, requested data is then prepared accordingly, anonymized, and made accessible together with the respective variable dictionary.

