# Supplementary material for: Effectiveness and cost-effectiveness of four different strategies for SARS-CoV-2 surveillance in the general population (CoV-Surv Study): a structured summary of a study protocol for a cluster-randomised, two-factorial controlled trial
Source: Trials. 2021 Jan 8;22:39. doi: 10.1186/s13063-020-04982-z (PMC7791150; doi:10.1186/s13063-020-04982-z)
Supplement: Supplementary file 1 — Additional file 1. [file 13063_2020_4982_MOESM1_ESM.pdf]

Studienprotokoll

Titel der Studie:

Cluster-randomisierte 2-faktorielle kontrollierte Studie zur Testung der (Kosten-)Effektivität von vier verschiedenen SARS-CoV-2 Surveillance-Strategien für die Allgemeinbevölkerung

Studienleiter: Dr. Andreas Deckert, Heidelberg Institute of Global Health, Im Neuenheimer Feld 324, Telefon: 06221-5637260, Email: a.deckert@uni-heidelberg.de

Weitere an der Studie Beteiligte:
Dr. Claudia Denkinger (Sektion Klinische Tropenmedizin, Universität Heidelberg)
Prof. Till Bärnighausen (Heidelberg Institute of Global Health)
Prof. Michael Marx (Heidelberg Institute of Global Health; evaplan GmbH)
Lucia Brugnara (evaplan GmbH)
Sandra Oerther (Heidelberg Institute of Global Health)
Prof. Michael Knop (Zentrum für Molekulare Biologie der Universität Heidelberg)
Dr. Simon Anders (Zentrum für Molekulare Biologie der Universität Heidelberg)
Prof. Manuela de Allegri (Heidelberg Institute of Global Health)
Dr. Hoa Nguyen (Heidelberg Institute of Global Health)
Dr. Aurélia Souares (Heidelberg Institute of Global Health)
Dr. Shannon McMahon (Heidelberg Institute of Global Health)
Dr. Tobias Siems (Institut für Angewandte Mathematik Heidelberg)
Lisa Koeppel (Sektion Klinische Tropenmedizin Heidelberg)
Dr. Rainer Schwertz (Gesundheitsamt Heidelberg)
Dr. Britta Knorr (Gesundheitsamt Heidelberg)
Dr. Andreas Welker (Gesundheitsamt Heidelberg)
Dr. Matthias Sand (GESIS Mannheim)

Biometriker:
Dr. Tobias Siems

Verantwortliche Studienärztin:
Dr. med. Claudia Denkinger

Datenbeauftragter:
Dr. Simon Anders

Unterstützende Institutionen, Firmen, Organisationen (finanziell/ideell):
Finanzierung durch das Bundesministerium für Bildung und Forschung

Datum & Version: 03.11.2020, Version V1.2

Inhaltsverzeichnis

1. Zusammenfassung ... 3
2. Einleitung/Wissenschaftliche Grundlagen ... 4
3. Ziele der Studie (allgemein) ... 6
4. Zielkriterien ... 7
5. Studienbedingte Maßnahmen / Studienablauf ... 8
6. Studientyp/Studiendesign ... 15
7. Einschlusskriterien ... 15
8. Ausschlusskriterien ... 15
9. Randomisierungsverfahren/-plan ... 15
10. Abbruchkriterien ... 16
11. Aufwandsentschädigung ... 16
12. Sicherheitslabor ... 16
13. Statistisches Design ... 16
14. Rechtliche und ethische Aspekte ... 24
15. Literaturverzeichnis ... 28

1. Zusammenfassung

Der Anfang der SARS-CoV-2-Pandemie in Deutschland war durch lokal entstandene Hotspots gekennzeichnet, der weiteren Ausbreitung wurde mit erheblichen Einschränkungen des täglichen Lebens und einem Lockdown im wirtschaftlichen Bereich begegnet. Inzwischen befinden wir uns in der zweiten Welle, die sich durch größere Hotspots auszeichnet und es steht zu befürchten, dass sich die Epidemie großflächig in Deutschland ausbreitet. Gleichzeitig ist ein weiterer Lockdown politisch kaum noch zu verkaufen.

Neben der sogenannten passiven Überwachung von Personen die mit COVID-19-Symptomen im Gesundheitswesen vorstellig werden und sich dann einem Test unterziehen, begrenzen sich viele aktive Teststrategien auf das Testen von bestimmten Bevölkerungsgruppen, wie z.B. Bewohner von Altenheimen, Mitarbeiter im Gesundheitswesen, Reiserückkehrer oder auch Schulen. Mit diesen Teststrategien werden auch asymptomatische Fälle entdeckt, sie dienen in der Regel allerdings nur der lokalen Eindämmung bzw. der Risikobegrenzung eines bereits etablierten Ausbruchs. Die genaue Anzahl von asymptomatischen SARS-CoV-2-Trägerne in der Bevölkerung bleibt dabei aber weiter im Dunkeln. Dies verhindert eine genauere Abschätzung der tatsächlichen Lage und die Möglichkeit von vorausschauenden Vorhersagen.

In dem vorliegenden Projekt sollen erstmals vier verschiedene aktive Surveillance-Strategien für die Allgemeinbevölkerung in einer cluster-randomisierten kontrollierten Studie in der Rhein-Neckar-Region (ca. 700.000 Einwohner) auf ihre Kosten-Effektivität getestet werden. Dazu wird eine Zufallsstichprobe von den Einwohnermeldeämtern der Rhein-Neckar-Region gezogen und auf die Studienarme aufgeteilt. Die Studienarme unterscheiden sich durch die Kombination zweier Faktoren: 1) Zufallsstichprobe von zufällig gezogenen heterogenen Einzelpersonen versus Zufallsstichprobe aus Personen innerhalb homogener Haushalte, 2) die direktes Testen versus Testen nach einem positiven Pre-Screening-Ergebnis. Für das Pre-Screening auf COVID-19-Symptome wird ein kurzer Papier- oder Online-Fragebogen verwendet.

Statt der herkömmlichen quantitativen Echtzeit PCR (qPCR) verwenden wir das Loop-Mediated Isothermal Amplification (LAMP) Verfahren zur Feststellung von infizierten Proben. Diese Methode hat ähnliche Test-Merkmale wie die qPCR, ist aber technisch einfacher und damit deutlich kostengünstiger und besser skalierbar. Außerdem werden wir statt Rachenabstrichen Speichelproben nehmen. Damit entfällt die Notwendigkeit der Beprobung durch medizinisches Personal und die Studienteilnehmer können die Speichelprobe selbst unkompliziert zuhause entnehmen. Die Logistik lässt sich damit relativ einfach über den Postweg abwickeln.

Die Studie wurde derart gestaltet, dass in allen vier Armen letztendlich die gleiche Anzahl von LAMP-Tests durchgeführt wird. Damit reflektieren wir die Situation von politischen Entscheidungsfindern, die basierend auf der beschränkten Ressource der Anzahl von verfügbaren Tests eine Entscheidung über die beste Strategie treffen müssen. Die Studie soll im November und Dezember durchgeführt werden, Ziel ist es in jedem Arm 2500 LAMP-Tests durchzuführen.

Nach der Feststellung der besten Strategie wird das System bei dann ggf. noch bestehendem Bedarf Anfang 2021 in eine Routine-Surveillance überführt. Mittels dieser Routine-Surveillance kann die Prävalenz der SARS-CoV-2-Infektionen in der Bevölkerung in einem gewissen Fenster monitoriert und auftretende Cluster gezielt lokal eingedämmt werden. Zudem wird mit dem Prävalenzschätzer ein prognostisches Model informiert, dass mehrwöchige Vorhersagen über den weiteren Verlauf und die zu erwartenden Kapazitätsengpässe im Gesundheitswesen erlaubt und damit frühe Gegenmaßnahmen ermöglicht, was einen weiteren Lockdown verhindern kann.

2. Einleitung/Wissenschaftliche Grundlagen

Das schwere akute respiratorische Syndrom COVID-19, verursacht durch das Coronavirus 2 (SARS-CoV-2) tauchte erstmals im Dezember 2019 in Wuhan, China, mit einer Häufung von Lungenentzündungen auf und hat sich seitdem über den ganzen Erdball verbreitet [1]. Zu den klinischen Merkmalen der Krankheit, die als COVID-19 bezeichnet wird, gehören Fieber, Kopfschmerzen und Husten, aber es wurden auch schwerere Symptome wie Atemnot und Atemstillstand gemeldet [2]. Bis zum 30. September wurden weltweit ca. 34 Millionen Erkrankungsfälle und mehr als 1.000.000 Todesfälle in 188 Ländern registriert [3]. Die rasche Eskalation der Situation veranlasste die Weltgesundheitsorganisation am 11. März 2020 eine Pandemie auszurufen [4].

Humane Coronaviren (HCoVs) sind dafür bekannt, dass sie 10-30% aller Infektionen der oberen Atemwege bei Erwachsenen verursachen, die meist folgenlos verlaufen [5]. In weniger als zwei Jahrzehnten traten jedoch zwei hoch pathogene Coronaviren, SARS-CoV und MERS (Middle East Respiratory Syndrome)-CoV, auf, die 2002 bzw. 2012 Epidemien auslösten [6]. Alle drei CoVs, SARS-CoV-2, SARS-CoV und MERS-CoV, wurden ursprünglich von Tier zu Mensch übertragen. Die anhaltende Übertragung von SARS-CoV-2 von Mensch zu Mensch ist jedoch die Hauptursache für die gegenwärtige Pandemie [7], die die Gesundheitssysteme und Labors des öffentlichen Gesundheitswesens vor enorme Herausforderungen stellt. Die genaue und rasche Identifizierung von infizierten Personen mit SARS-CoV-2 ist daher der Schlüssel für eine sofortige klinische Versorgung und für die unverzügliche Einführung von Maßnahmen des öffentlichen Gesundheitswesens zur Eindämmung der Krankheitsausbreitung.

Für die drastischen Maßnahmen, die beim Ausbruch der Pandemie im Frühjahr 2020 in Deutschland und anderen Ländern ergriffen wurden, war vor allem die Auslastung der Kapazität der Intensivbetten in den Krankenhäusern maßgebend. Eine Überlastung dieser Kapazitäten hätte auch in Deutschland erhöhte Todesraten zur Folge gehabt. Aufgrund der Ausbreitungs-Muster von SARS-CoV-2 ist eine komplette Eindämmung der Pandemie in naher Zukunft kaum möglich. Deshalb kann die Pandemie bis zum Vorhandensein eines effektiven Vakzins, einer möglichen natürlichen Abschwächung der Virulenz des Erregers oder dem Erreichen von Herdenimmunität so kontrolliert werden, dass die Kapazitäten des Gesundheitswesens nicht überlastet und zugleich weitere drastische Maßnahmen verhindert werden können. Zudem hat ein übermäßiges Freihalten von Bettenkapazitäten negative Auswirkungen bei anderen Krankheiten.

Die aktuellen Strategien zur Aufdeckung neuer Fälle von COVID-19 in Deutschland stützen sich auf die passive Überwachung von symptomatischen Patienten oder Risikogruppen sowie der Kontrolle von Krankenhausaufnahmen, damit überwiegend späte Marker erfasst. Diese Maßnahmen eignen sich allerdings nur bedingt, asymptomatische Träger zu erfassen und die Pandemie aufbauend auf der tatsächlichen Prävalenz vorausschauend zu kontrollieren; zumal die Symptome bei mildem Verlauf sehr ähnlich zu einer normalen Erkältung mit anderen Viren (z.B. Rhinoviren) sind und deshalb häufig nicht diagnostiziert werden. Es gibt jedoch neuere Belege dafür, dass asymptomatische SARS-CoV-2-Infizierte eine ähnliche Viruslast tragen wie symptomatische Patienten, was darauf hindeutet, dass auch Personen ohne oder mit minimalen Symptomen das Potenzial haben, die Erkrankung zu übertragen [8]. Es wird vermutet, dass asymptomatische Träger um die 40% aller Infektionen mit SARS-CoV-2 ausmachen [9]. Die Überwachung von Veranstaltungen und Risikokonstellationen wie sie in anderen Arbeitsgruppen des Netzwerkes der Universitätsmedizin geplant ist, wird helfen, Super-Spreader zu identifizieren und das Übertragungsrisiko in gewissen Gruppen zu minimieren. Für eine dauerhafte Kontrolle der Infektions-Prävalenz innerhalb eines gewissen verkraftbaren Fensters ist jedoch die komplementäre Überwachung der Allgemeinbevölkerung notwendig. Nur so können

Infektionsherde früh erfasst und eine realistische Abschätzung der Gesamtprävalenz vorgenommen werden. Diese Schätzung des nicht diagnostizierten und des asymptomatischen Anteils von SARS-CoV-2-Infizierten ist entscheidend, um die tatsächliche Krankheitslast in der Bevölkerung zu ermitteln.

Ein aktives Surveillance-System für die Allgemeinbevölkerung nimmt mit dem Verlauf der Pandemie an Bedeutung zu. Während am Anfang von einer niedrigen Gesamtprävalenz in der Allgemeinbevölkerung aber von einer hohen Prävalenz in lokalen Clustern ausgegangen werden kann, wird sich die Infektion im Laufe der Zeit allgemeiner verbreiten und zu einer langsam steigenden Gesamtprävalenz in der Allgemeinbevölkerung führen, da zum einen drastische Maßnahmen wie ein kompletter Lockdown gelockert werden und zum anderen im Vergleich dazu weniger effektive Maßnahmen wie das Tragen von Masken zwar starke lokale Ausbrüche verhindern aber die Verbreitung nicht komplett unterbinden können.

Die passiven Strategien müssen daher durch eine aktive und engmaschige Routineüberwachung größerer Bevölkerungsgruppen mit regelmäßigen Tests ergänzt werden. Bei positiven Testresultaten müssen diese Personen kontaktiert, isoliert und deren persönliches Umfeld auf weitere Infektionen getestet werden können, damit die Verbreitung des Virus unterbunden wird. Dabei ist zu beachten, dass mit dem Öffnen des öffentlichen Lebens die Testfrequenz enorm erhöht werden muss, um größere Infektionsketten und die sogenannten Super-Spreader Events zu verhindern (oder zumindest detektieren), die einen erneuten Shutdown erzwingen würden. Die aus einer solchen Routineüberwachung der Bevölkerung abgeleiteten Pandemie-Parametern ermöglichen eine vorausschauende Abschätzung des weiteren Verlaufs durchgeführt und erlauben einen gezielten und effektiven Einsatz lokaler und kurzfristiger Gegenmaßnahmen.

Da COVID-19 eine Atemwegserkrankung ist, die ähnlich übertragen wird wie saisonale Viruserkrankungen, kann erwartet werden, dass insbesondere zur Grippesaison 2020/2021 sowohl die Infektionsraten von SARS-CoV-2 zunehmen werden. Gleichzeitig wird aber auch eine Zunahme von COVID-19 ähnlichen Erkrankungssymptomen zu verzeichnen sein, wodurch die Dichte und Häufigkeit von SARS-CoV-2 Tests noch gesteigert werden müssen. In einer idealen Welt würde eine aktive Überwachung jede einzelne Person häufig testen, um alle asymptomatischen und symptomatischen Träger frühestmöglich aufzuspüren und die Weiterverbreitung zu unterbinden. Es liegt auf der Hand, dass eine solche Strategie unrealistisch ist und wir werden daher mit zeitlich variierenden cluster-randomisierten Stichproben arbeiten. Die Größenordnung dieser Stichproben erlaubt die Abschätzung der Prävalenz unter Berücksichtigung der Unsicherheit.

Der derzeitige Goldstandard für den Nachweis von SARS-CoV-2 ist die Methode der reversen Transkriptase-Polymerase-Kettenreaktion (RT-PCR), eine Technologie, die auf dem Nachweis viraler RNA aus respiratorischen Proben beruht [10]. Der RT-PCR-Nachweis ist sehr sensitiv und erlaubt den Virus-RNA-Nachweis während einer akuten Infektion und auch bis zu 2-3 Wochen nach der Infektion. Gegenwärtig sind die Kosten für eine RT-PCR im Bereich von 25-40€ pro Analyse, ohne Berücksichtigung der Kosten für die Probengewinnung. Damit für eine aktive Überwachung mittels Zufallsstichproben die notwendigen Testgrößen in einem vernünftigen finanziellen Rahmen analysiert werden können, müssten die Rachenabstriche allerdings gruppiert analysiert werden (gepooltes Testen) [11]. Dabei reduzieren sich die Sensitivität des Nachweises proportional zur Größe der Testpools. Zusätzlich dazu stellt das Einsammeln von Nasopharyngealen oder Oropharyngelaen Abstrichen (Rachenabstriche) eine zusätzliche logistische Herausforderung dar, da für solche Probengewinnung medizinisch geschultes Personal und entsprechende Ausrüstung (infektionsschutz) benötig werden.

Als Alternative bieten sich Proben an, welche die Testpersonen selber gewinnen können und welche einfach transportiert werden können. In diesem Zusammenhang wurden in den letzten 2-3 Monaten

mehr und mehr Studien vorgestellt, die viele Belege liefern, dass Speichel, Gurgellösung und Hustenschleim (eine Art Sputum) geeignete Quelle von Patientenmaterial für den sensitiven Nachweis von SRAS-CoV-2 darstellen [12, 13, 14, 15]. Eine Auflistung und Diskussion der Literatur und Preprints zu diesem Thema finden sich auch auf der Seite des RKI [16]. Um die Kosten für den molekularbiologischen Nachweis der viralen RNA in diesen Proben zu reduzieren, eignet sich auch eine isothermale RNA Amplifikation basierte Methode (RT-LAMP). Nachdem diese Methode anfänglich noch weniger sensitiv für den Nachweis von viraler RNA war [17], sind für diese Methode inzwischen auch neue DNA Primersets entwickelt worden, die einen ähnlich sensitiven Nachweis der viralen RNA ermöglichen wie mittels RT-PCR [18, 19]. Der große Vorteil an RT-LAMP ist, dass die Methode einfacher durchzuführen ist und im Gegensatz zur RT-PCR keinen Thermocycler benötigt und dass die Reagenzien deutlich kostengünstiger sind.

Inzwischen sind die Probengewinnung und SARS-CoV-2 Diagnose mittels einer Kombination aus Gurgel- und Speichelprobe die auch Komponenten der oberen Atemwege enthält (im Folgenden pauschal ‚Speichelprobe' genannt) und mittels RT-LAMP in Heidelberg durch Arbeiten der AG Knop, Anders und DaoThi sehr gut etabliert. In diesen Proben werden ähnliche Virenlasten gemessen wie in nasopharyngealen Abstrichen. Wichtig ist, dass die Speichelprobe den Instruktionen entsprechend entnommen wird. Zudem stellt sich immer mehr heraus, dass Speichel sehr stabil ist und dass sich die Virenlast im Speichel auch im Verlauf von mehreren Tagen und bei Lagerung bei Raumtemperatur (20-25°C) kaum verändert (Resultate der AG Knop, Anders und DaoThi, und auch [20]), so dass sich der Transport von Speichelproben mittel der Post anbietet. Dadurch ergeben sich insgesamt sehr viel günstigere Verfahren bei denen Selbst-Beprobung mittels Speichel, Transport mittels Post und RNA Nachweis durch RT-LAMP die bisher viel teureren pharyngealen Abstriche und RT-PCR basierte Detektion ersetzen können, so dass Surveillance-Studien in den Bereich des (finanziell) Möglichen geraten.

3. Ziele der Studie (allgemein)

Mit dieser cluster-randomisierten 2-faktoriellen kontrollierten Studie sollen vier verschiedene SARS-CoV-2 Bevölkerungs-Surveillance-Strategien getestet werden. In allen vier Armen der Studie werden jeweils 2500 Speichelproben von zufällig ausgewählten freiwilligen Teilnehmern/Teilnehmerinnen eingesammelt und mittels RT-LAMP (ggf. gefolgt von einem Bestätigungstest mittels RT-PCR im Fall von positiven Proben) auf eine vorliegende SARS-CoV-2-Infektion getestet (detaillierte Beschreibung siehe Kapitel 13). Die erfolgreiche Strategie soll später in ein Routine-Surveillance-System überführt werden, das auch für andere Pandemien in kurzer Zeit einsatzbereit wäre. Die aus dem Surveillance-System abgeleiteten gleitenden Prävalenzschätzer werden Vorhersagen zum weiteren regionalen Verlauf der Pandemie und zur Auslastung des Gesundheitssystems im Rhein-Neckar-Raum erlauben. Dazu benutzen wir einen Bayesschätzer, der die Daten sequentiell und rückblickend auf ein begrenztes Zeitfenster verarbeitet.

Nach der Erprobung der besten Strategie werden alle Ressourcen vollständig für die Routineüberwachung zur Verfügung stehen, was die Genauigkeit der Prävalenz-Schätzer erhöhen wird. Durch solch eine Routine wird ermöglicht, abhängig vom Verlauf der Pandemie, bis zu 1000 Speichelproben pro Tag zu sammeln; daraus ergeben sich 5000 LAMP-Tests pro Arbeitswoche. Diese Daten werden uns helfen, auf kritisch steigende Infektionszahlen zu reagieren, insbesondere wenn die Inzidenz ein unerwartet starkes Wachstum aufweist.

Der quantitative Teil der Studie wird durch qualitative Komponenten ergänzt. Dabei werden Interviews mit einer kleinen Gruppe mit einem kleinen Gruppen von Teilnehmer*innen der Studie aber auch mit

Non-Respondern geführt, um Barrieren für die Teilnahme, Herausforderungen bei der Teilnahme, Einstelllungen gegenüber dem Testen und Gründe für die Teilnahme oder Nicht-Teilnahme zu erfahren. Zusätzlich wird eine kleine Gruppe von Studienteilnehmer*innen gebeten, nach der Studienteilnahme an einer kleinen Umfrage teilzunehmen und über Ihre Erfahrungen mit dem Abgeben der Speichelprobe und der Teilnahme zu berichten. Die dabei gewonnenen Erkenntnisse sollen bei der Überführung in ein Routine-System berücksichtigt werden.

4. Zielkriterien

4.1. Hauptzielkriterien

1) Kosten pro richtig gescreenter Person
2) Kosten pro positiven Fall
3) Prävalenz
4) Präzision der Prävalenz-Schätzung

4.2. Nebenzielkriterien

1) Teilnahmerate
2) Kosten pro asymptomatischen Fall
3) Bevölkerungsprävalenz
4) Anzahl asymptomatischer Fälle pro Arm
5) Verhältnis symptomatische zu asymptomatischen Fällen pro Arm

Mittels zusätzlicher Interviews/Fragebögen (alters- und geschlechtsstratifizierte Zufallsauswahl aus allen ursprünglich kontaktierten Personen):

6) Durchschnittlicher Zeitaufwand für die Teilnahme
7) Teilnehmerzufriedenheit

4.3. Zusätzliche Untersuchungen in der Studie (nicht Teil des Versuchs):

1) Kosteneffektivität jeder der vier Überwachungsstrategien im Vergleich zur reinen passiven Überwachung (d.h. Status Quo)
2) Erarbeitung eines statistischen Tools zur Vorhersage der Krankenhausauslastung durch SARS-CoV-2
3) Zeit von der Testversendung bis zur Testanwendung und Zeit von Testversendung bis zum Testergebnis
4) Qualitative Interviews: Wahrnehmung und Präferenzen der zu Testenden in Bezug auf die Teststrategien

Erläuterungen:

Kosten pro richtig gescreenter Person: Kosten der Prozesse i) Kontaktieren, ii) Pre-screening (Arm B1, B2) und iii) biologischer Test für alle Personen; Nenner: alle Personen die richtige vollständig gescreent wurden, also i) beim Pre-screening mit negativem Ergebnis mitgemacht und daher keinen biologischen Test erhalten haben, ii) beim Pre-Screening mit positivem Ergebnis mitgemacht und daher auch einen biologischen Test durchgeführt haben.

Kosten pro positiven Fall (asymptomatisch und symptomatisch): Gesamtkosten aller Personen geteilt durch die Anzahl derjenigen die im biologischen Test (nach Bestätigungstest) richtig-positiv sind.

Prävalenz: 4-wöchige kumulative SARS-CoV-2 Prävalenz; hier gilt der Arm A1 als Gold-Standard für die Schätzung, die Studie erlaubt die Messung der Verzerrung in den anderen Armen; falls einer dieser Arme in Routine gehen sollte, kann dort entsprechend korrigiert werden.

Präzision der Prävalenz: Konfidenzintervalle der Schätzer in jedem Arm

Kosten pro asymptomatischen Fall: Gesamtkosten aller Personen geteilt durch die Anzahl derjenigen, die keine Symptome zeigen und trotzdem richtig positiv getestet wurden (Arm A1, A2, B2).

Teilnahmerate: Anzahl der Personen für die ein biologisches Testergebnis (Arm A1, A2) bzw. ein Pre-Screening und ggf. ein biologisches Testergebnis vorliegt (Arm B1, B2) geteilt durch die Anzahl der Personen die kontaktiert wurden.

Teilnehmerzufriedenheit: gemessen mithilfe der Likert-Skala

5. Studienbedingte Maßnahmen / Studienablauf

5.1. Studienbedingte Maßnahmen

Im Rahmen dieser Studie werden die Teilnehmer*innen gebeten, eine Speichelprobe durch Gurgeln mit 5ml Kochsalzlösung abzugeben. Diese Probe wird mittels RT-LAMP im Labor auf SARS-CoV-2 analysiert. Die darin enthaltenen positiven Proben werden zur abschließenden Abklärung einer RT-PCR unterzogen. Die positiven Fälle werden dem Gesundheitsamt zur weiteren Abklärung und zur bereits etablierten Kontaktverfolgung gemeldet. Vom Gesundheitsamt erfasste positive Kontakte werden an die Studie zurückgemeldet, um zu sehen, ob der Erstkontakt Teil eines Clusters war. Die Teilnehmer werden gebeten, für die Kontaktaufnahme durch das Gesundheitsamt ihre Telefonnummer oder Email-Adresse anzugeben.

Ein Teil der Teilnehmer*innen nimmt vor Abgabe der Speichelprobe an einem online- oder papierbasierten Pre-Screening Teil. Es werden Fragen zu COVID-19 spezifischen frühen Symptomen gestellt. Der Fragensatz umfasst ca. 10-15 Fragen, ergänzt um soziodemografische Angaben (Bildungsabschluss, Erwerbshintergrund, Haushaltsgröße). Die Teilnehmer*innen müssen die Speichelprobe nur abgeben, wenn ein bestimmter Schwellenwert im Pre-Screening überschritten wird.

Der andere Teil der Teilnehmer*innen wird gebeten, freiwillig Angaben zu COVID-19 spezifischen Symptomen zu machen. Hier findet die Testung der Speichelprobe unabhängig von den Symptomen statt (Details siehe Kapitel 13).

Qualitative Teilstudie: Eine kleine Gruppe von Teilnehmer*innen und Non-Respondern wird nach ihrem individuellen Studienabschluss zudem gebeten, an Interviews teilzunehmen. Zusätzlich wird eine weitere kleine Gruppe gebeten, an einer qualitativen Umfrage mit offenen Fragen teilzunehmen.

5.2. Rekrutierung und Aufklärung

Die potenziellen Studienteilnehmer*innen werden von den Einwohnermeldeämtern des Studiengebiets nach dem Zufallsprinzip ausgewählt (Details siehe Kapitel 13.2). Identifizierte potenzielle Studienteilnehmer*innen ab einem Alter von 7 Jahren werden dann initial per Post kontaktiert. Das weitere Vorgehen unterscheidet sich im Detail hinsichtlich der vier zu testenden verschiedenen Strategien:

Strategie A1: Einzelpersonen aus der Zufallsstichprobe erhalten eine schriftliche Anfrage zur Studienteilnahme, zusammen mit einer genauen Beschreibung der Studie und deren Abläufe, Kontaktdaten inklusive der Telefonnummern einer Hotline im Gesundheitsamt sowie den Materialien

zur Selbst-Beprobung, sowie einem frankierten Rückumschlag und Hinweisen wie die Proben zurückgeschickt bzw. an Sammelstellen abgegeben werden können. Die Studieninformation klärt auch allgemein über das weiterhin bestehende Risiko bei negativem Testergebnis und über die Konsequenzen eines positiven Tests auf. Die Informationsmaterialien werden verständlich und übersichtlich gehalten, für detaillierte Informationen wird auf die Studien-Website und die Hotline verwiesen. Das Röhrchen für das Probenmaterial enthält einen Aufkleber mit einer eindeutigen Identifikationsnummer (ID) als Barcode.

Das Anschreiben enthält auch einen Link zu einem Online-Video, das in einfacher Sprache und bildhaft die Probenentnahme demonstriert. Ebenfalls enthalten ist eine Einwilligungserklärung, die zusammen mit der Probe zurückgesandt werden soll. Die Einwilligungserklärung enthält den Barcode für die Identifikation des Teilnehmers/der Teilnehmerin, das Datum des Versands und freie Felder für den Eintrag des Datums und der Uhrzeit der Probenentnahme sowie der Telefonnummer oder Email-Adresse.

Zusammen mit der Einwilligungserklärung und der Speichelprobe kann zusätzlich freiwillig ein Kurz-Fragebogen zum Allgemeinbefinden und zu COVID-19-Symptomen ausgefüllt und mit zurückgeschickt werden.

Falls von den Teilnehmern/Teilnehmerinnen innerhalb von max. 4 Tagen nach Versand des Schreibens keine Speichelprobe vorliegt, wird ein Erinnerungsschreiben versandt.

<u>Strategie A2:</u> Die Vorgehensweise ist hier wie bei A1, nur dass hier die zufällig gezogenen Einzelpersonen aufgefordert werden, Speichelproben aller Familien-/Haushaltsmitglieder abzugeben. Es werden pauschal Materialien für 3 Haushaltsmitglieder verschickt. In dem Anschreiben wird darauf hingewiesen, dass weitere Proben-Röhrchen für zusätzliche Haushaltsmitglieder über die Hotline angefordert werden können. Die Einwilligungserklärung enthält hier eine separate Zeile für jedes Haushaltsmitglied für die Angabe von Namen, Geburtsdatum und biologischem Geschlecht sowie für die Unterschrift, um die Einwilligung zu bestätigen. Die freiwillige Angabe von COVID-19 Symptomen ist für alle Haushaltsmitglieder ebenfalls möglich.

<u>Strategie B1:</u> Diese Strategie beinhaltet die Anwendung eines SARS-CoV-2 spezifischen Symptom-Screenings vor der Selbst-Beprobung. Die zufällig gezogenen Teilnehmer*innen werden in einem ersten Schreiben über die Studie und deren Abläufe informiert (inklusive des weiteren Vorgehens und potentieller Konsequenzen, s. A1) und aufgefordert, an einem Online-Pre-Screening (Smartphone-, Tablet-, und Computer-kompatibel) teilzunehmen. Die Einwilligung zur Teilnahme erfolgt online; bei Minderjährigen erfolgt sowohl die Einwilligung als auch das Symptom-Screening durch den/die Erziehungsberechtigte(n). Das Schreiben enthält einen Link und einen QR-Code mit einer eindeutigen ID, die das Ergebnis des Pre-Screenings mit den Teilnehmerdaten verbindet. Falls die Teilnehmer*innen innerhalb von 48 Stunden nach Versand des Schreibens nicht an dem Online-Pre-Screening teilgenommen haben, wird ein Erinnerungsschreiben verschickt. Das Erinnerungsschreiben enthält das Pre-Screening in Form eines Papierfragebogens (inklusive schriftlicher Einwilligung) mit einem frankierten Rückumschlag. Auf diese Weise werden auch Personen erreicht, die keinen Zugang zum Internet haben.

Bei einem positiven Pre-Screening-Ergebnis erhalten die Teilnehmer*innen ein weiteres Schreiben mit der weiteren Vorgehensweise wie bei A1 (leicht abgeänderte Materialien, da die Studienaufklärung schon früher erfolgte).

<u>Strategie B2:</u> Wie A2, nur dass hier wieder komplette Haushalte von positiv detektierten Personen Speichelproben abgeben sollen.

Die Studienteilnehmer*innen können die Testergebnisse über die eindeutige ID online abfragen. Bei Vorliegen eines positiven LAMP-Tests wird dem Teilnehmer/der Teilnehmerin mitgeteilt, dass ein Bestätigungstest (PCR) noch aussteht. Sollte der Bestätigungstest positiv ausfallen, wird der Teilnehmer vom Gesundheitsamt kontaktiert. Das Gesundheitsamt führt dann eine Routine-Kontaktverfolgung durch. Die Ergebnisse aus der Kontaktverfolgung werden zur Identifizierung von Clustern vom Gesundheitsamt pseudonymisiert abgefragt.

Die versandten Studienmaterialien und die Materialien zur Selbst-Beprobung werden im Vorfeld in einer qualitativen Studie zur Benutzerfreundlichkeit und Akzeptanz untersucht. Dafür wird gesondert ein Ethikvotum eingeholt.

Die Studienmaterialien werden dreisprachig in Deutsch, Türkisch und Englisch verfasst. Zusätzlich wird eine Hotline am Gesundheitsamt eingerichtet, die mit 3-5 Personen ebenfalls mehrsprachig besetzt ist.

Die Studie wird im Vorfeld durch umfangreiche Öffentlichkeitsarbeit (Presse, Radio, Internet, Social Media, Aushänge) bekannt gemacht. Die Veröffentlichung dieser Materialien erfolgt in enger Abstimmung mit der Task Force des Universitätsklinikums Heidelberg sowie mit der Koordinierungsstelle für das nationale COVID-19 Forschungsnetzwerk der Universitätsmedizin in Berlin und dem Gesundheitsamt Rhein-Neckar.

Auf Incentives oder Teilnahmeanreize z.B. in Form einer Lotterie wird bewusst verzichtet, da diese Werkzeuge in einem Routine-System auch nicht zur Verfügung stehen und somit die Ergebnisse verzerren würden.

5.3. Rekrutierung und Aufklärung qualitative Teilstudie

Für die qualitative Komponente werden für die Durchführung von Interviews potentielle Teilnehmer*innen zufällig nach Abschluss der Studienteilahme bzw. aus dem Pool der Non-Responder gezogen und telefonisch oder postalisch kontaktiert. Für die Studienteilnehmer*innen liegen Telefonnummern bzw. Email-Adressen aus der Einwilligungserklärung zur Kontaktaufnahme vor. Bei den Non-Respondern wird zunächst versucht, die Telefonnummern anhand der Adressdaten in öffentlich zugänglichen Datenbanken zu recherchieren und einen Kontakt über Telefon herzustellen. Ist dies nicht möglich, erhält die Person eine schriftliche Einladung zur Teilnahme an dem qualitativen Teil der Studie. Ziel ist es, insgesamt maximal 15 Teilnehmer*innen des Corona-Tests und 15 Non-Responder sowie 25 asymptomatische Coronavirus-Träger zu interviewen. Die Rekrutierung endet vorzeitig, falls sich eine Sättigung in den Antworten der Teilnehmer*innen abzeichnet (Details siehe Kapitel 13.4). Für die Interviews werden getrennte Informationsmaterialien und eine separate Einwilligungserklärung verwendet.

Zusätzlich werden zufällig Personen aus den Teilnehmer*innen der quantitativen Studie nach dem individuellen Studienabschluss ausgewählt und mit einem Anschreiben eingeladen, Ihre Erfahrungen in einem Online-Survey oder mittels des beiliegenden Fragebogens mitzuteilen (Details siehe Kapitel 13.4). Die Rekrutierung endet wenn 100 Antworten vorliegen oder vorzeitig, falls sich eine Sättigung in den Antworten der Teilnehmer*innen abzeichnet. Auch hierzu werden getrennte Informationsmaterialien und eine separate Einwilligungs-erklärung verwendet.

5.4. Studienablauf/Zeitaufwand

Von Mitte Oktober bis Mitte November erfolgt die Ziehung der Einwohnermeldestichprobe in den beteiligten Gemeinden, entsprechend dem Randomisierungsplan (s. Kapitel **Error! Reference source not found.**). Die Anfrage an die Einwohnermeldeämter sowie die anschließende Adresslieferung wird

ca. 4 Wochen dauern, so dass mit dieser Arbeit aufgrund der Dringlichkeit des gesamten Projektes schon vor Erhalt des finalen Ethikvotums begonnen wird. Die Daten verbleiben bis zur Klärung des Ethikvotums beim Gesundheitsamt Rhein-Neckar. Falls keine abschließende Genehmigung durch die Ethikkommission erteilt werden sollte, werden diese Daten umgehend vernichtet.

Das Einsammeln der Speichelproben soll von Mitte November bis maximal Ende Dezember erfolgen. Die gezogene Gesamtzahl der Adressen wird dazu zunächst auf 4-Wochen-Chargen aufgeteilt, die dann nacheinander abgearbeitet werden, wobei an jedem Tag Anschreiben verschickt werden. Jede der vier Chargen beinhaltet eine unabhängige, zweistufige Stichprobenziehung der Adressen, bei der zuerst Sampling Points (Cluster) und anschließend eine Personenstichprobe des Einwohnermeldeamtes gezogen wird (siehe hierzu Kapitel 13.2). Die Nachverfolgung von Personen, die erst auf die Erinnerungsschreiben reagieren kann sich dabei mit dem Beginn einer neuen Wochen-Charge überlappen.

Nach Ziehen der Einwohnermeldestichprobe im gesamten Studiengebiet erfolgt der Versand der Anschreiben/Studienmaterialien über das Gesundheitsamt Rhein-Neckar. Die Logistik dazu wird zentral am Zentrum für Molekulare Biologie der Universität Heidelberg gehalten.

In den Armen A1 und A2 sollen die Teilnehmer*innen sofort eine Speichelprobe abgeben. Der Zeitaufwand für das Lesen der Materialien und das Anschauen des Online-Videos beträgt ca. 10-15 Minuten. Die Speichelprobe sollte nach Möglichkeit vor dem Frühstück und vor dem Putzen der Zähne entnommen werden. Der Zeitaufwand für die Entnahme der Probe beträgt ungefähr 5 Minuten. Hinzu kommt ein gewisser Zeitaufwand für das Zurücksenden der Probe (Einpacken, Einwurf Briefkasten) oder das Abgeben der Probe an Sammelstellen (z.B. Sammelbehälter im Rathaus).

In den Armen B1 und B2 werden die Teilnehmer*innen aufgefordert, an einem Online- bzw. papierbasierten Fragebogen-Screening teilzunehmen. Der Zeitaufwand für das Lesen der Studienmaterialien beträgt ca. 10-15 Minuten, der Zeitaufwand für das Pre-Screening selbst beträgt ca. 10 Minuten. Der Zeitaufwand für die Abgabe der Speichelprobe nach einem positiven Pre-Screening-Ergebnis entspricht A1 und A2.

Insgesamt ist der Zeitaufwand für die Teilnehmer*innen in A2 und B2 ein wenig höher, da diese Teilnehmer*innen gebeten werden, Speichelproben aller Haushaltsmitglieder mit einzusammeln. Der Zeitaufwand pro Speichelprobe bleibt aber der gleiche.

5.5. Zeitaufwand für die qualitative Teilstudie

Die Teilnahme an den Interviews erfordert einen Zeitaufwand von maximal 1 Stunde. Die Teilnahme an dem Survey zu den Erfahrungen in der Studie erfordert maximal 20 Minuten Zeitaufwand.

5.6. Art und Quelle des Biomaterials

Testdurchführung

Ein wichtiger Punkt der Studie beinhaltet, dass die Proben von den Teilnehmer*innen selber zu Hause entnommen werden. Dadurch wird eine extrem aufwändige Logistik mit einer Probenentnahme unter hohen Sicherheitsvorkehrungen durch geschultes medizinisches Personal vermieden. Die Proben werden zurückgeschickt oder an zentralen Sammelpunkten gesammelt und am Testzentrum in der Virologie und des Zentrums für Molekulare Biologie prozessiert und ausgewertet.

Die Anleitungen, wie eine Speichelprobe genau entnommen und das Teströhrchen zurückgeführt werden soll, erhalten die Teilnehmer*innen über das Internet und anhand eines bereitstehenden Instruktionsvideos bzw. eines Flyers. Entsprechend der Anleitung im Instruktionsvideo entnehmen sich

die Teilnehmer*innen dann zu Hause eine Speichelprobe und verfahren mit der Probe wie im Video angegeben. Es wird auch erklärt, wie das Teströhrchen zurückzuführen ist. Dabei gibt es zwei Möglichkeiten: entweder per Post zurückgeschickt oder durch Abgabe an einer ausgewiesenen Sammelstelle. Für beide Möglichkeiten sind genaue Instruktionen gegeben. Diese sind auch über die Test-Webseite bzw. telefonisch unter Angabe der ID abrufbar.

Für die Testdurchführung werden vorbereitete Teströhrchen verwendet, welche über einen Barcode verfügen, der die Zuordnung erlaubt. Die Proben werden in Laboren der Virologie und des Zentrums für Molekulare Biologie der Universität analysiert. Für die Probenverarbeitung wird die RT-LAMP-basierte Methodik angewendet um die virale RNA zu amplifizieren [17]. Im Falle eines positiven LAMP-Testes wird dann eine RT-qPCR Analytik im Diagnostiklabor des Uniklinikums Heidelberg erfolgen, um die Diagnose zu bestätigen. Für die den Einsatz dieses Testverfahrens liegt bereits ein positives Votum der Ethikkommission Heidelberg vor (S-392/2020).

Im Rahme der Studie wird die Rachenspülwasser wie folgt verwendet:

- Wir werden Loop-mediated Isothermal Amplification (LAMP) Assays benutzen: Für diesen Assay werden entweder die Proben direkt oder es wird daraus isolierte RNA verwendet. Die Proben werden bei 85-95°C für 5 bis 30 min erhitzt und direkt in den LAMP Reaktionsmix gegeben oder mit anderen geeignete Extraktionslösungen behandelt; die isolierte RNA wird direkt in den LAMP Reaktionsmix gegeben. Die Reaktion wird bei 65°C inkubiert und danach analysiert. Zur Analyse kommen etablierte Methoden zum Einsatz: Fluoreszenzmessung, Gelelektrophorese, Farbumschlag oder LAMP-Seq Methoden [17].
- Proben in denen ein positiver Nachweis von viraler RNA erfolgt ist, werden dann mittels RT-qPCR analysiert, um den Nachweis unabhängig zu erbringen. Dazu wird ein Set an Primern verwendet um einen großen Bereich des viralen Genoms zu amplifizieren und zu sequenzieren. Damit soll festgestellt werden, ob Mutationen im viralen Genom vorliegen. Dies dient der Kontrolle, um festzustellen, ob SARS-CoV-2 Stämme im Umlauf sind, die nicht mehr mit allen für die SARS-CoV-2 verwendeten Primer detektiert werden können.

5.7. Lagerung der Proben

DNA-Analysen der humanen DNA in den Proben werden nicht durchgeführt. Die Proben werden nach Vorliegen des Testergebnisses vernichtet.

5.8. Datenmanagement/Datenschutzkonzept

Angemessene Maßnahmen zur Gewährleistung des Datenschutzes und der Vertraulichkeit werden von den Studienprüfern gebührend berücksichtigt. Nationale Vorschriften zum Datenschutz werden befolgt, und es werden keine Informationen über persönliche Daten von Spendern oder Patienten weitergegeben, es sei denn, eine solche Weitergabe ist für die Durchführung von Überwachungsverfahren (z.B. Meldung von positiven Trägern ans Gesundheitsamt), die Pharmakovigilanz oder die Rückverfolgbarkeit zum Schutz der öffentlichen Gesundheit unerlässlich.

Das Datenmanagement, die Berichterstattung und die Datenspeicherung im Rahmen dieser Studie werden vollständig den Anforderungen der ICH Good Clinical Practice entsprechen. Die Daten werden lokal eine elektronische Datenbank am Universitätsklinikum Heidelberg eingegeben. Die Daten werden auf einem sicheren, passwortgeschützten Server gespeichert und regelmäßig gesichert. Das Online-Pre-Screening-Tool und die damit zusammenhängende Datenbank sind ebenfalls auf einem Server der Universität untergebracht. In die Datenverwaltung werden Qualitätskontrollmechanismen eingebaut.

Alle Daten, die während dieser Studie gesammelt werden, werden für einen Zeitraum gespeichert, der für die daraus abgeleiteten aktuellen und zukünftigen Pandemie-Forschungsfragen bestimmt wird.

Die Kontaktdaten des Datenschutzbeauftragten des Universitätsklinikums Heidelberg lauten:
Universitätsklinik Heidelberg
Datenschutzbeauftragte
Im Neuenheimer Feld 672, 69120 Heidelberg, Germany
Tel: +49 6221 56 7036, E-mail: datenschutz@med.uni-heidelberg.de

Die Namen der Teilnehmer*innen und alle anderen vertraulichen Informationen unterliegen der ärztlichen Schweigepflicht und den Bestimmungen der Datenschutz-Grundverordnung (DSGVO) sowie des Landes- bzw. Bundesdatenschutzgesetzes (LDSG bzw. BDSG). Positiv getestete SARS-CoV-2-Träger werden entsprechend dem Infektionsschutzgesetz pseudonymisiert an das Gesundheitsamt übermittelt. Das Gesundheitsamt kann dann über eine sichere verschlüsselte Schnittstelle mittels der übermittelten Identifikationsnummer die Klarnamen, Telefonnummern und/oder Email-Adressen aus der Datenbank auslesen. Für die Ermittlung von Clustern werden vom Gesundheitsamt die Anzahl weiterer positiv getesteter Kontakte erfragt. Die daraus abgeleiteten Kartierungen erfolgen pseudonymisiert in einem Maßstab, der Rückschlüsse auf einzelne Haushalte nicht ermöglicht. Eine darüberhinausgehende Weitergabe von Patienten-/Probandendaten erfolgt ggf. nur in pseudonymisierter Form. Dritte erhalten keinen Einblick in Originalunterlagen.

Für weitergehende statistische Untersuchungen werden die Daten wie folgt pseudonymisiert: Name, Adresse und Geburtsdatum werden durch eine pseudonyme ID ersetzt, lediglich Alter in Jahren, Geschlecht, und Postleitzahl werden beibehalten, sowie eine pseudonyme Haushalts-ID, die erlaubt, zu erkennen, ob zwei Datensätze aus demselben Haushalt stammen, Die Zuordnung von IDs zu Primärdaten wird verschlüsselt, der Schlüssel verbleibt bei demjenigen, der die Pseudonymisierung durchgeführt hat.

5.9. Studienbeginn und Studiendauer

Im Rahmen der Studie sollen insgesamt 10.000 Speichelproben eingesammelt werden. Wir planen die Studie von Mitte November bis Mitte Dezember durchzuführen. Die Studie läuft solange, bis alle vier Wochen-Chargen an zufällig gezogenen Adressen abgearbeitet sind. Damit wird die Chancengleichheit der Gemeinden bzgl. der Teilnahme gewährleistet. Abhängig von den tatsächlichen Response-Raten kann es dadurch zu einer unterschiedlichen Gesamtzahl an Speichelproben in den Armen kommen, was bei der Ergebnisauswertung berücksichtigt wird.

Die qualitative Teilstudie startet parallel zur Hauptstudie. Die Durchführung der Interviews kann nach Ende der Hauptstudie solange fortgeführt werden bis genügend Interviewdaten gesammelt sind. Dies gilt auch für den Survey zu den Teilnahme-Erfahrungen.

5.10. Überführung in ein Routine-Surveillance-System

Nach Ablauf der Studie soll gegen Ende 2020 eine umfassende Bewertung der vier Strategien erfolgen und die geeignete Strategie bei Bedarf in ein Routine-System überführt werden. Dieses Routine-System soll entsprechend der aktuellen Lage auf bis zu 1.000 Speichelproben pro Tag skalierbar sein. Das Routine-System soll nach der SARS-CoV-2-Pandemie in einen Stand-By-Modus übergehen und jederzeit bei neuen Pandemie-Bedrohungen schnell wieder aktivierbar sein. Die Verwendung der LAMP-Methode erlaubt dabei eine rasche Umstellung der Test-Methodik auf andere Erreger.

5.11. Belastung/Risiken

Pre-Screening-Tool: Den Teilnehmern/Teilnehmerinnen entsteht durch die Teilnahme am Pre-Screening kein zusätzliches Risiko. Teilnehmer*innen am Pre-Screening könnten eventuell durch die Abfrage der Symptome verunsichert werden. Wir richten eine Hotline ein, um verunsicherten Teilnehmern/Teilnehmerinnen eine Anlaufstelle zu bieten.

Probenentnahme: Die Probenentnahme ist nicht invasiv, da kein zusätzliches Gewebe oder Blut entnommen werden. Daher ist der Zeitaufwand für die Teilnehmer*innen gering und es besteht kein zusätzliches Risiko. Wir haben die Abnahmeprozedur getestet und einen Zeitaufwand von ca. 10 bis 15 Minuten bestimmt. Im Wiederholungsfall (wenn ein*e Teilnehmer*in einen zweiten Test macht) ist der benötigte Zeitaufwand nur noch ca. 3-5 Minuten. Eine Verletzungsgefahr besteht bei der Probenentnahme nicht.

Probenversand: Die Speichelproben werden in Rückumschlägen postalisch zurückgeführt oder an Sammelstellen abgegeben. Die verwendeten Rückumschläge entsprechen den Sicherheitsanforderungen für den postalischen Versand von Biomaterialien, so dass eine Gefährdung der Allgemeinheit bei einem Leck ausgeschlossen werden kann. Die Anforderungen für den Versand von potentiell infektiösen Biomaterialien werden berücksichtigt.

Interviews und Survey: Den Teilnehmer*innen entsteht durch die Teilnahme an den Interviews und im Survey kein zusätzliches Risiko. Fühlt sich ein(e) Teilnehmer(in) bei gestellten Fragen unwohl, so kann er/sie die Antwort verweigern bzw. das Interview oder die Teilnahme am Survey jederzeit abbrechen. Bereits erhobene Daten werden auf Verlangen gelöscht und nicht bei der Auswertung berücksichtigt.

5.12. Wirkung/Nutzen

Eigennutzen: Durch das LAMP Hochdurchsatz-Screening können SARS-CoV-2 Träger früh identifiziert werden und isoliert werden. Falls der Träger schon Symptome entwickelt haben sollte, kann er dann direkt medizinisch behandelt werden. Die Testergebnisse werden online abrufbar sein, positiv getestete Personen werden zusätzlich vom Gesundheitsamt kontaktiert und entsprechende Maßnahmen eingeleitet.

Gruppennutzen: Das direkte Umfeld von positiv getesteten Personen wird ebenfalls unmittelbar vom Gesundheitsamt kontaktiert und auf SARS-CoV-2 untersucht. Damit wird eine Ausbreitung einer Infektion in der direkten Umgebung von Teilnehmern eingegrenzt.

Durch die qualitative Teilstudie werden Erkenntnisse gewonnen, die helfen können, die Akzeptanz des Testansatzes zu erhöhen und damit indirekt die Detektionsraten von Coronavirus-Trägern. Außerdem ermöglicht die qualitative Teilstudie einen gewissen partizipatorischen Einfluss der Betroffenen auf die Implementierung eines Routine-Systems.

Fremdnutzen: Ein Surveillance-System mit zufällig gezogenen Teilnehmern aus der Bevölkerung erlaubt es, die Pandemie in einem gewissen Prävalenz-Fenster zu überwachen und einzudämmen. Dadurch wird der Einsatz von „harten“ Gegenmaßnahmen wie z.B. ein erneuter Shutdown verhindert und gleichzeitig die Funktion der Gesundheitsversorgung aufrechterhalten. Zudem können entstehende Cluster frühzeitig entdeckt und lokal eingegrenzt werden. Durch die Freiwilligkeit der Teilnahme und der abgeleiteten gewissen Sicherheit für die Region entstehen eine gewisse Akzeptanz und damit auch ein Nutzen für die gesamte Bevölkerung.

6. Studientyp/Studiendesign

Es handelt sich um eine cluster-randomisierte, 2-faktoriell kontrollierte, prospektive, interventionelle, einfachverblindete (verblindete Test-Auswertung) Studie mit Forschung an Biomaterialien.

7. Einschlusskriterien

7.1. Hauptstudie

Eingeschlossen wird folgender Personenkreis:

- Probanden/Probandinnen ab 7 Jahre
- Vorliegende Online bzw. alternativ papierbasierte informierte Einwilligungserklärung für die Teilnahme am Online- bzw. Fragebogenscreening
- Vorliegende papierbasierte Einverständniserklärung für die Verwendung der Speichelprobe und der Daten

7.2. Qualitative Teilstudie

- Teilnehmer*innen sowie Non-Responder der Hauptstudie ab 18 Jahren
- Vorliegende telefonische bzw. alternativ papierbasierte oder elektronische (Interview: Email; Survey: online tool) Einwilligungserklärung für die Teilnahme am Interview bzw. am Survey
- Vorliegende telefonische bzw. alternativ papierbasierte oder elektronische (Interview: Email; Survey: online tool) Einverständniserklärung für die Verwendung der Daten

8. Ausschlusskriterien

8.1. Hauptstudie

- Nichterfüllung der Einschlusskriterien
- Entzug der Einwilligung

8.2. Qualitative Teilstudie

- Nichterfüllung der Einschlusserklärungen
- Entzug der Einwilligung

9. Randomisierungsverfahren/-plan

Aufgrund des zweistufigen Ziehungsverfahrens der Gemeinden (außerhalb Heidelbergs) erfolgt die Abfrage der Adressen, die eine Gemeinde liefern soll, insgesamt über alle vier Arme hinweg. Die von den Einwohnermeldeämtern gezogenen Zufallsstichproben werden entsprechend der benötigten Adressen in den jeweiligen Armen im Verhältnis 5:2,5:14:7 zufällig den Armen A1, A2, B1, B2 zugeteilt. Die Anzahl der von den Einwohnermeldeämtern angeforderten Adressen ergibt sich als Summe der Adressen, die sich aus den vier unabhängig gezogenen, zweistufigen Stichproben der jeweiligen Chargen ergeben. Da sich die Auswahl und Anzahl der Sampling Points pro Gemeinde in jeder dieser vier Chargen unterscheiden kann, entstehen Variationen in der Gesamtzahl der Adressen (über alle vier Chargen hinweg) pro Gemeinde. Von einer a priori Stratifizierung (bspw. nach Alter und Geschlecht) wird aufgrund des Ziehungsverfahrens (siehe Kapitel 13.2) sowie der daraus

resultierenden Problematik gering- oder unbesetzter Ziehungszellen abgesehen. Die Zuteilung auf die jeweiligen Erhebungsarme erfolgt unter der Verwendung uneingeschränkter Zufallsstichproben. Die Anzahl der kontaktierten Adressen pro Arm bleibt für alle vier Chargen konstant. Aufgrund der unabhängigen Ziehung pro Charge variiert jedoch die Anzahl der Kontaktadressen pro Gemeinde. Um Probleme der Ganzzahligkeit zu vermeiden, wird der Cox-Algorithmus für kontrolliertes Runden angewendet [21].

10. Abbruchkriterien

Individuelle Abbruchkriterien

Studienteilnehmer*innen, denen die Bereitstellung der Speichelprobe Ekel bereitet oder die es nicht schaffen, eine Speichelprobe bereitzustellen, können die Studie jederzeit abbrechen.

10.1. Abbruchkriterien für die Gesamtstudie

Die Gesamtstudie wird abgebrochen falls sich die SARS-CoV-2 Pandemie-Situation entsprechend verschärft und es früh absehbar ist, dass die Studie keinen Mehrwert liefert, sondern woanders dringend benötigte Ressourcen bindet. Dies kann die Testkapazität betreffen, aber auch Engstellen beim Gesundheitsamt.

11. Aufwandsentschädigung

Die Teilnehmer*innen erhalten keine Aufwandsentschädigung. Es sind allerdings Verlosungen unter allen Speichelproben-Spendern zur Erhöhung der Teilnahmebereitschaft geplant.

12. Sicherheitslabor

Alle Proben werden in Laboren der Sicherheitsstufe 2 verarbeitet.

13. Statistisches Design

Die Fragestellung welche die Entscheidungsfindung von Regierungen wiederspiegelt lautet: Welche Strategie ist die (Kosten-)effektivste, gegeben einer vorgegebenen Anzahl von vorhandenen Tests (externe Kapazitätsbeschränkung). Die Stichprobengröße für jeden Arm wurde daher derart bestimmt, dass die erwartete Anzahl von Tests in allen Armen gleich hoch sein würde, gegeben bestimmter Annahmen über die Teilnahmequoten und die Sensitivität und Spezifität des Pre-Screening Tools. Wir haben diesen Ansatz gewählt, weil die verbindlichen Randbedingungen und der Haupttreiber der Kosten pro Person der tatsächlich durchgeführte Test sind. Damit ist die Vergleichbarkeit der Prävalenz, der Rate der vollständig gescreenten Personen und zusätzlich die Vergleichbarkeit der (Kosten-)Effektivität gewährleistet.

Das Studienende ist erreicht, wenn in allen vier Surveillance-Strategien alle vier Wochen-Chargen und Adressen abgearbeitet und die dabei eingesammelten Speichelproben gesammelt und auf aktuelle SARS-CoV-2-Infekionen getestet wurden. Damit werden Verzerrungen vermieden, die sich aufgrund schwer erreichbarer Personen/Haushalte ergeben können, die erst verzögert antworten. Demnach ist es möglich, dass die Netto-Fallzahl in Abhängigkeit der Rücklaufquote ober- oder unterhalb der 2500 Speichelproben liegt. Dies wird in der Auswertung entsprechend berücksichtigt.

Für die vier verschiedenen Strategien werden zwei Faktoren kombiniert: 1) Eine Zufallsstichprobe von zufällig gezogenen heterogenen Einzelpersonen versus einer Zufallsstichprobe aus Personen innerhalb homogener Haushalte, 2) die direkte Selbst-Beprobung versus der Selbst-Beprobung nach einem positiven Pre-Screening-Ergebnis. Der Ansatz der Selbst-Beprobung ganzer Haushalte beruht dabei auf der Annahme, dass die Wahrscheinlichkeit für mehrere positive Personen innerhalb von Haushalten bei Vorhandensein einer positiven Person erhöht ist.

	Ohne Pre-Screening	Mit Pre-Screening
**Einzelpersonen**	Arm A1	Arm B1
**Familien/Haushalte**	Arm A2	Arm B2

Abbildung 1: 2-faktorielles Design

Mit Strategie A1 wird theoretisch ein Abbild der tatsächlichen SARS-CoV-2-Prävalenz in der Bevölkerung gemessen, da asymptomatische und symptomatische SARS-CoV-2-Träger gleichermaßen erfasst werden. Falls innerhalb von homogenen Haushalten eine höhere Ansteckungswahrscheinlichkeit besteht, könnte sich in Arm A2 die Rate der SARS-CoV-2-Träger erhöhen und zusätzlich initiale Cluster auf Haushaltsebene detektiert werden. In Arm B1 werden komplett asymptomatische SARS-CoV-2-Träger durch das Vorschalten eines Pre-Screening-Tools, welches frühe COVID-19-Symptome abfragt, ausgeschlossen. Allerdings werden für die finalen 2500 Speichelproben in diesem Arm ursprünglich deutlich mehr Menschen angeschrieben und erreicht. In Arm B2 geben alle Familien-/Haushaltsmitglieder einer positiv gescreenten Person eine Speichelprobe ab, so dass in diesem Arm die Wahrscheinlichkeit asymptomatische SARS-CoV-2-Träger zu detektieren zwischen der Wahrscheinlichkeit in den Armen A2 und B1 liegt. Das Ablaufdiagramm der Strategien ist in Abbildung 2 dargestellt. Die berichteten Werte entsprechen der angestrebten Nettofallzahl.

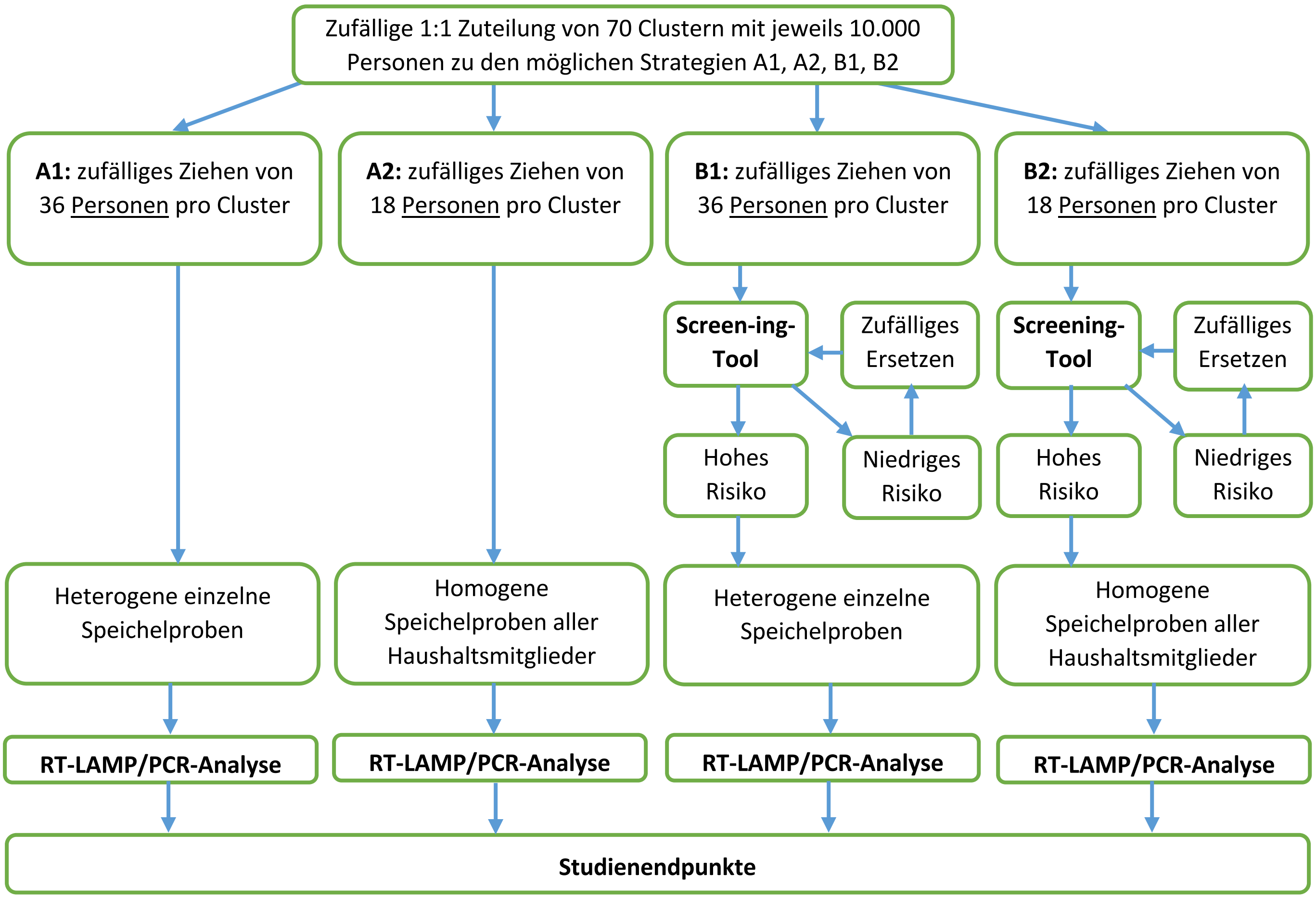

Abbildung 2: Studien-Ablaufdiagramm

13.1. Fallzahl

Im Routine-Betrieb soll die erfolgreiche Strategie mit bis zu 1000 Speichelproben pro Tag mittels eines gleitenden Bayesianischen Prävalenzschätzers 2- bis 5-wöchige Vorhersagen zur kritischen Auslastung der Intensivbettenkapazität im Rhein-Neckar-Raum und/oder anderer Parameter der Gesundheitsversorgung erlauben und frühe lokal begrenzte Eindämmungsmaßnahmen ermöglichen [22].

Die Bewertung der besten Strategie kann nicht von einem einzigen Parameter abhängig gemacht werden, es werden mehrere Parameter in einem iterativen Prozess in eine umfassende Bewertung einfließen. Als Ausgangspunkt sollen alle Strategien eine gewisse Prävalenz mit ausreichender Genauigkeit schätzen können. Zum Zeitpunkt der Studienplanung im Mai 2020 lag die 4-wöchige kumulative COVID-19 Prävalenz im Rhein-Neckar-Raum bei ca. 0,25%. Geht man von einer ebenso hohen Prävalenz an asymptomatischen SARS-CoV-2-Trägern aus, betrug die Gesamtprävalenz von asymptomatischen und symptomatischen SARS-CoV-2-Trägern ca. 0,5%. Die aktuell steigenden Fallzahlen lassen vermuten, dass im November ein ähnliches Prävalenz-Szenario wie im Mai vorliegen könnte. Die Zahl von 2500 Speichelproben ermöglicht damit in Strategie A1, die als reine Zufallsstichprobe das Pandemie-Geschehen direkt abbilden sollte, die Detektion einer Gesamtprävalenz von 0,5% mit einer Präzision von ±0,35% mit einer Power von 95% (Konfidenzinterval nach Agresti-Coull [23]); eine Prävalenz von 0,2% mit einer Genauigkeit von ±0,25% kann mit einer Power von 93% erfasst werden. Falls die Annahme einer höheren Prävalenz von SARS-CoV-2 Infektionen in Familien/Haushalten mit infizierten Personen zutrifft, wird in Strategie A2 eine höhere Detektionsrate erwartet. In den Armen B1 und B2 mit vorgeschaltetem Pre-Screening-Tool sollte die Detektionsrate durch die Filterwirkung des Tools ebenfalls anders ausfallen. Ausgangslage für einen Vergleich der Strategien ist der Erhalt einer Detektionsrate der Größenordnung wie in Arm A1. Darauf aufbauend werden (Kosten-)Effektivitätsaspekte in die Bewertung der Strategien mit einbezogen.

Eine Fallzahlplanung für eine klassische Überlegenheitsstudie erscheint aus mehreren Gründen schwierig. Unter anderem unterliegt der Verlauf der SARS-CoV-2-Prävalenz einer dynamischen Entwicklung die aktuell noch keine Voraussagen für die Situation im November erlaubt. Durch die Studie wird erstmals eine genaue Abschätzung der Prävalenz und der Dynamiken ermöglicht, diese fließen dann zusammen mit der (Kosten-)Effektivitätsanalyse in eine Gesamtbewertung der Strategien ein.

Die final ausgewählte Strategie wird im Routine-Betrieb durch die freiwerdenden Ressourcen aus den anderen Studien-Armen genauere Prävalenzschätzer liefern. Falls eine Strategie ausgewählt werden sollte, die die repräsentative Prävalenz aus Arm A1 überschätzt, wird eine entsprechende Anpassung des Bayesianischen prognostischen Modells vorgenommen. Strategien mit überschätzter Gesamt-Prävalenz bieten den Vorteil, Cluster schneller zu detektieren und die Kontaktverfolgung zu intensivieren.

Die Stichprobenziehung muss so gestaltet werden, dass letztendlich in jedem Arm der Studie 2500 Speichelproben für die Analyse vorliegen. Einzugsgebiet der Studie ist der Rhein-Neckar-Raum einschließlich Heidelberg mit ca. 700.000 Einwohnern. Um eine hinreichende Präzision der Schätzung zu erreichen, wird daher eine Gesamtnettofallzahl von 1,4 % der Zielpopulation bzw. 0,35 % (netto) pro Erhebungsarm angestrebt. Für die unterschiedlichen Arme der Studie ergibt sich dann die notwendige Stichprobengröße wie folgt:

Arm A1 (zufälliges Ziehen von Einzelpersonen, direkte Zusenden der Materialien zur Selbst-Beprobung): Bei einer angenommenen Response von 50% (inklusive Erinnerungsschreiben) sind hier 5000 Adressen/Anschreiben über 4-6 Wochen notwendig.

Arm A2 (zufälliges Ziehen von Einzelpersonen, direktes Zusenden der Materialien zur Selbst-Beprobung der angeschlossenen Familien/Haushalte): Die durchschnittliche Familiengröße in Deutschland beträgt ca. 2 [24]. Unter dieser Annahme und ebenfalls einer Response von 50% (inklusive Erinnerungsschreiben) müssten hier 2500 Einzelpersonen angeschrieben werden.

Arm B1 (zufälliges Ziehen von Einzelpersonen, Versenden der Materialien zur Selbst-Beprobung nach positivem Pre-Screening-Ergebnis): Hier werden insgesamt 3 Anschreiben verschickt, 1) Anschreiben inklusive Link/QR-Code zu Online-Pre-Screening Tool, 2) Erinnerungsschreiben mit Papierfragebogen, 3) Anschreiben mit Materialien zur Selbst-Beprobung. Wir gehen davon aus, dass der Rücklauf bei denjenigen die am Online-Pre-Screening teilgenommen haben und ein positives Pre-Screening-Ergebnis erhalten 80% beträgt. Damit in diesem Arm insgesamt 2500 Speichelproben vorliegen, müssen daher 3125 Testkits versandt werden. Bei einer Sensitivität von 90% und einer Spezifität von 70% des Pre-Screening-Tools und einer SARS-CoV-2 Prävalenz von 0,5% müssen 3125/0,303=10313 Personen am Pre-Screening teilnehmen. Sollte die Spezifität des Pre-Screening-Tools niedriger als 70% sein, wird die notwendige Zahl der Speichelproben mit weniger Personen erreicht, da die Anzahl der falsch positiv gescreenten Personen steigt. Sollte die Sensitivität nieder als 90% ausfallen, fällt das bei einer Prävalenz von 0,5% kaum ins Gewicht. Unter der Annahme von 50% Rücklauf im ersten Anschreiben und weiteren 50% Rücklauf im Erinnerungsschreiben müssen damit insgesamt 13750 Personen initial angeschrieben werden.

Arm B2 (zufälliges Ziehen von Einzelpersonen, Versenden der Materialien zur Selbst-Beprobung an die angeschlossenen Haushalte nach positivem Pre-Screening-Ergebnis im Haushalt): wie B1, allerdings hier Haushalte mit durchschnittlich 2 Mitgliedern. Damit müssen 6875 initial angeschrieben werden.

Insgesamt sind damit für die Durchführung der 4-armigen Studie mit den oben genannten Annahmen 28125 Adressen notwendig.

13.2. Stichprobenziehung

Die Ziehung der Adressstichprobe erfolgt stratifiziert nach den beiden Schichten „Heidelberg“ und der Schicht „Region Rhein-Neckar“ getrennt und unter der Anwendung unterschiedlicher Stichprobendesigns. Entsprechend der Bevölkerungsanteile der beiden Schichten an der gesamten Zielpopulation werden Schichtgewichte größenproportional berechnet und die Gesamtnettofallzahl von 10.000 Adressen anhand dieser auf die beiden Schichten aufgeteilt. Um Probleme der Ganzzahligkeit zu vermeiden, wird ein statistischen Runden unter der Verwendung des Cox-Algorithmus angewandt. Im Anschluss wird die Nettostichprobengrüße anhand der Überlegungen der unterschiedlichen Ausschöpfungsquoten nach Untersuchungsarm in Kapitel 13.2 auf die entsprechende Bruttostichprobe übersetzt. Dadurch ergeben sich insgesamt 6.386 zu kontaktierende Adressen in Heidelberg sowie 21.739 zu kontaktierende Adressen in der Schicht „Rhein-Neckar-Kreis“. Die Anzahl wird im Anschluss gleichmäßig und unter der Verwendung von statistischem Runden auf die vier Chargen aufgeteilt.

Da die Gemeinde Heidelberg die Einzige innerhalb ihrer Schicht ist, erfolgt die Adressauswahl in einem einstufigen Ziehungsvorgang, da diese Gemeinde somit sicher in der Stichprobe enthalten ist.

Für die Schicht „Region Rhein-Neckar“ wird für jede Charge ein zweistufiges Stichprobendesign angewendet, innerhalb derer die primären Erhebungseinheiten (Primary Sampling Units (PSU)) als synthetische Cluster und als sekundäre Erhebungseinheiten (Secondary Sampling Units (SSU))

Adressen gezogen werden. Die Auswahl der PSUs erfolgt unter der Verwendung des sog. pps-Samplings, bei der die Ziehungswahrscheinlichkeit größenproportional bestimmt wird. Pro Charge werden 55 PSUs unabhängig gezogen. Zur Ermittlung der Ziehungswahrscheinlichkeit wird das Verhältnis der Populationsgröße innerhalb einer Gemeinde zur Populationsgröße innerhalb der Schicht verwendet. Auf zweiter Stufe erfolgt eine disproportionale Ziehung, bei der für jede PSU die gleiche Anzahl von SSUs gezogen wird. Das in dieser Schicht angewendete Ziehungsverfahren wird auch oftmals als sog. selbstgewichtender Ansatz bezeichnet, bei der sich aufgrund dieses Vorgehens (theoretisch) die gleichen Ziehungswahrscheinlichkeiten für jede Adresse in der Schicht ergeben [25].

Eine Anzahl von 55 PSUs wird gegeben der Anzahl der Gemeinden und unter Berücksichtigung des Intra-Class-Korrelationskoeffizenten als geeignet erachtet. Diese 55 PSUs werden dann unter der Verwendung des Cox-Algorithmus auf die 56 Gemeinden (Sampling Points) in der Region Rhein-Neckar aufgeteilt. Dabei ist es möglich, dass ein Sampling Point mehrere PSUs beinhaltet.

Zugleich wird bei Bedarf unter der Anwendung des K-Nearest-Neighbor-Algorithmus strukturähnliche Gemeinden ermittelt, die für den Fall einer Verweigerung auf Gemeinde-Ebene als Substitut herangezogen werden können [26]. Pro Sampling Point werden maximal 3 Substitute ermittelt. Aufgrund des hohen Verhältnisses zwischen zu ziehenden PSUs und der Anzahl der Gemeinden innerhalb dieser Schicht, wird davon abgesehen, bereits in der Stichprobe gezogene Gemeinden als Substitut zu sperren. Mit dem Ausfall einer Gemeinde kann es dadurch möglich sein, dass dies zu einem Anstieg der zu ziehenden Adressen einer anderen, in der Stichprobe enthaltenen Gemeinde führt.

Innerhalb der PSU wird im Anschluss die Anzahl der SSUs gezogen. Pro PSU wird dabei die gleiche Anzahl an Adressen nachgefragt. Da ein Sampling Point (Gemeinde) mehrere PSUs enthalten kann, ist es durchaus möglich, dass die Anzahl der Adressen pro Gemeinde (und Charge) variiert. Die gezogenen Adressen pro Gemeinde werden, wie bereits in Kapitel 9 erläutert, im Anschluss zufällig auf die vier Studienarme im Verhältnis 5:2,5:14:7 aufgeteilt. Die Anzahl der innerhalb einer Gemeinde abgefragten Adressen ergibt sich aus der Summe der Adressen pro Gemeinde und Charge. Da jeder Charge eine unabhängige, zweistufige Stichprobenziehung (innerhalb der Schicht „Rhein-Neckar-Kreis") mit 55 PSUs zugrunde liegt, kann sich die Gesamtzahl der Adressen pro Gemeinde voneinander unterscheiden-

13.3. Statistische Analysen

Unsere primäre Analyse wird die Intent-to-treat-Analyse (ITT) sein. In sekundären Analysen werden wir auch die durchschnittlichen kausalen Effekte (complier average causal effect) schätzen, indem wir die anfängliche zufällige ITT-Zuweisung zu den vier Armen unserer Studie als instrumentelle Variable (IV) verwenden und die Ergebnisse mit Hilfe von Standard-IV-Analysen analysieren [27].

Die Kosten für die Bereitstellung der verschiedenen Screening-Dienstleistungen werden für jede Person als kontinuierliche Variable gemessen. Die Effekte auf kontinuierliche primäre und sekundäre Endpunkte - Kosten pro vollständig gescreenter Person, Kosten pro bestätigtem positivem Fall, Kosten pro asymptomatischem Fall, Präzision der Prävalenz, durchschnittliche Zeit, die jeder Teilnehmer für die Teilnahme aufgewendet hat (erhoben in einer Untergruppe) - werden unter Verwendung der Standardregression der kleinsten Quadrate (OLS) gemessen. Für die Auswirkungen auf die binären Endpunkte - Prävalenz und Beteiligung – werden wir das modifizierte Poisson-Modells verwenden [28]. Schließlich werden wir die Effekte auf den geordneten kategorialen Endpunkt - die Teilnehmerzufriedenheit, gemessen mit der Likert-Skala - mit Hilfe einer geordneten logistischen Regression messen.

In allen unseren Analysen werden wir die Clusterbildung von Standardfehlern auf der Ebene der Randomisierungseinheit, d.h. der Gemeinde, berücksichtigen. In unseren primären Analysen werden wir keine Basislinien-Kovariablen berücksichtigen; in sekundären Analysen werden wir die Basislinien-Kovariablen Alter und Geschlecht berücksichtigen.

Mikrokostenrechnung und Kostenwirksamkeitsanalyse

Um eine umfassende Bewertung zu ermöglichen und die optimalste Überwachungsstrategie zu ermitteln, werden wir die oben genannten ITT-Analysen durch eine Mikrokostenrechnung und eine Kostenwirksamkeitsanalyse ergänzen, in denen die Kosten der vier Überwachungsstrategien und ihre Kostenwirksamkeit im Verhältnis zum Status quo eines nur passiven Überwachungssystems bewertet werden.

Da die rechtzeitige und genaue Erkennung sowohl asymptomatischer als auch symptomatischer Covid-19-Patienten für die Kontrolle der Übertragung in der Gemeinschaft von entscheidender Bedeutung ist, wird die Studie aus der Perspektive des Gesundheitssystems durchgeführt, d.h. es werden alle Kosten einbezogen, die auf der Ebene des Gesundheitssystems anfallen. Bei der Kostenrechnung und der Kostenwirksamkeitsanalyse werden die wirtschaftlichen Kosten aller Ressourcen berücksichtigt, die im Zusammenhang mit der Umsetzung der Überwachungsstrategien verbraucht werden. Insbesondere werden wir die Kosten im Zusammenhang mit der Einführung und Aufrechterhaltung der vier aktiven Überwachungsstrategien sowie die Kosten des passiven Überwachungssystems als Vergleichsgröße berücksichtigen.

Um die Kosten der vier Überwachungsstrategien abzuschätzen, wird ein aktivitätsbasierten/Micro-Costing-Ansatz angewandt und prospektiv Daten zum Ressourcenverbrauch aller Aktivitäten im Zusammenhang mit der Umsetzung der vier Strategien gesammelt. Die Aktivitäten werden in zwei aufeinanderfolgende Phasen (Start und Implementierung) eingeteilt. Für jede Aktivität werden wir Daten zum Ressourcenverbrauch sowohl für Kapitalkostenposten (z.B. Gebäude, Ausrüstung und Fahrzeuge) als auch für wiederkehrende Kostenposten (z.B. Personal, Material und Verbrauchsmaterialien) direkt aus den Versuchsaufzeichnungen gewinnen. Daten zu den Stückkosten erhalten wir aus sekundären Datenquellen, wie z.B. Jahresabschlüsse der durchführenden Stellen, Rechnungen, Gehaltslisten. Wir werden die Daten zum Ressourcenverbrauch mit den Daten zu den Einheitskosten kombinieren, um die durchschnittlichen Kosten pro vollständig gescreenter Person, Kosten pro bestätigtem positivem Fall, Kosten pro asymptomatischem Fall, die für jede Strategie ermittelt wurden, zu schätzen.

Um die Kostenwirksamkeit jeder der vier Überwachungsstrategien im Vergleich zum Status quo zu bewerten, werden die Kosten und die Auswirkungen (gemessen als Anzahl der bestätigten positiven Fälle) jeder Strategie in Beziehung zu ihrem Komparator, dem rein passiven Überwachungssystem, gesetzt. Die von den Hauptzielkriterien erhaltenen Kosten- und Effektschätzungen werden in die Kostenwirksamkeitsanalyse einfließen. Darüber hinaus werden die Kosten und die Auswirkungen des passiven Überwachungssystems auf der Grundlage einer Kombination verschiedener sekundärer Datenquellen (z.B. Anzahl der getesteten Fälle und Anzahl der im Kontrollbereich entdeckten Fälle) geschätzt, die von den lokalen Gesundheitsbehörden und aus anderen Quellen (z.B. Erstattungssatz für RCR) zu erhalten sind. Wir werden die inkrementelle Kosten-Effektivitäts-Relationen (ICERs) für jede Strategie separate berechnen, d.h. die inkrementellen Kosten für jeden zusätzlichen Fall, der festgestellt wird und die für jeden der vier Überwachungsstrategien gegenüber dem Status quo vorliegen.

Informationen darüber, welche Überwachungsstrategie die niedrigere ICER ergibt, werden in Verbindung mit Informationen über die Modellierung bewertet, um die Entscheidungsfindung bei der

Wahl der am besten geeigneten Überwachungsstrategie, die in der Routinepraxis umgesetzt werden soll, zu unterstützen.

Prognostisches Modell

Auf Grundlage aktueller Hospitalisierungszahlen und den Testergebnisses aus den einzelnen Studienarmen sollen Vorhersagen bezüglich der Krankenhausauslastung gemacht werden. Dies ermöglicht ein rechtzeitiges und insbesondere lokales Eingreifen in die Krankheitsausbreitung.

Hierzu legen wir eine SEIHR (suspectible, exposed, infectious, hospitalized, removed) Differentialgleichung zugrunde. Einige der Parameter dieses Modells werden direkt aus der Literatur entnommen. Andere, insbesondere die für die Studie wichtigen wie die Basisreproduktionszahl und die Hospitalisierungsrate, werden durch einen Bayesianischen Ansatz modelliert.

Um der stark vereinfachten Darstellung des SARS-CoV-2 Infektionsverlaufs durch unser Modell gerecht zu werden, ziehen wir für die Vorhersagen jeweils nur ein begrenztes Zeitfenster an Daten heran. Dies ermöglicht dem Model, flexibler auf Änderungen im Krankheitsverlauf zu reagieren.

Das Inferenzmodel wird jeweils an die Studienarme angepasst und Simulationsstudien sollen dann die Vorhersagequalität der einzelnen Arme aufzeigen. Hierbei ist von Interesse, in welchem Zeitraum ein erneuter Ausbruch mit akzeptabler Wahrscheinlichkeit detektiert werden kann.

Schlägt das Vorhersagemodell in der Praxis einmal Alarm, kann dann lokal auf den Ausbruch reagiert werden. Die Dringlichkeit der Maßnahmen lässt sich durch die Anzahl der detektierten Infizierten in den Regionen, aber auch durch Indikatoren bezüglich des Ansteckungspotentials bestimmen. Letzteres lässt sich indirekt durch den CT Wert erkennen, aber auch durch Betrachtung der durch das Kontakt-Tracing gesammelten Daten.

13.4. Analysen der qualitativen Teilstudie: Wahrnehmung und Präferenzen der zu Testenden in Bezug auf die Teststrategien

Die qualitative Komponente, die in den Hauptstudie eingebettet ist, wird in drei Teile gegliedert.

Teil 1. Erfahrungen mit der Aufnahme versus Ablehnung der Studienteilnahme

Diese qualitative Teilstudie konzentriert sich auf die Perspektiven der Testdurchführung bzw. -abnahme, indem mit denjenigen gesprochen wird, die gezielt aus der breiteren quantitativen Studie ausgewählt wurden und Verhaltensweisen zeigen, die von Interesse sind (Aufnahme vs. Abnahme).

Perspektiven von Akzeptanten und Ablehnern des SARS-CoV-2-Tests		
Übergeordnetes Ziel	Untersuchung der sozial konstruierten Bedeutungen und Folgen der Durchführung oder Ablehnung eines SARS-CoV-2-Tests während der zweiten Welle der laufenden Pandemie	
Befragte Gruppe	• Diejenigen, die am Pre-Screening teilgenommen haben<br>• Diejenigen, die eine Speichelprobe abgegeben haben	• Diejenigen, die nicht am Pre-Screening teilgenommen haben<br>• Diejenigen, die keine Speichelprobe abgegeben haben
Größe der Stichprobe	Bis zu maximal 15 Personen jeweils aus den Armen A1 und A2 zusammen und B1 und B2 zusammen	Bis zu maximal 15 Personen jeweils aus den Armen A1 und A2 zusammen und B1 und B2 zusammen
Stichprobenziehung	Zweckmäßige Ziehung aus der quantitativen Studienstichprobe mit Repräsentativität von Geschlechtern und Altersgruppen	
Methode	Ausführliches Interview	

Hauptfragestellung	Was hat Sie dazu bewogen, an der Studie teilzunehmen?	Was hat Sie dazu bewogen, an der Studie nicht teilzunehmen?
Nebenfragestellungen	• Was halten Sie von Corona-Tests und Tests im Allgemeinen (und in Bezug auf deren Verwendung, Bedeutung und Konsequenzen in Ihrem Leben)?	
	• Was halten Sie speziell von der Methode der Speichelprobe zur Durchführung eines Corona-Tests?	
	• Was bedeutet ein positives oder negatives Testergebnis für Sie in Ihrem Leben?	
Rückfragen	Praktikabilität, Wahrnehmungen/ Erfahrungen der Testmodalitäten, Komfort, Erwünschtheit, Erfahrung mit der Selbst-Beprobung und dem Empfang von Testergebnissen, Wahrnehmung der Konsequenzen (Risiko der Quarantäne), Rolle der Fürsorge und Liebe für andere Familienmitglieder, kollektive oder soziale Verantwortung	Risikowahrnehmung, Kosten und Nutzen eines Corona-Tests, Rolle der Fürsorge und Liebe für andere Familienmitglieder, kollektive oder soziale Verantwortung

Tabelle 1: Zusammenfassung der Interviews zur Aufnahme und Ablehnung der Studienteilnahme

Teil 2. Asymptomatische SARS-CoV-2-Träger

Diese qualitative Teilstudie wird sich auf die Perspektiven derjenigen konzentrieren, die einen bestätigten, positiven SARS-CoV-2-Status haben, aber asymptomatisch sind.

Perspektiven asymptomatischer SARS-CoV-2-Träger	
Übergeordnetes Ziel	Erforschung der Art und Weise wie Männer und Frauen, bei denen eine asymptomatische SARS-CoV-2-Infektion diagnostiziert wurde, ihrer Diagnose Bedeutung verleihen und ebenso der Dialektik zwischen dem gleichzeitigen Gefühl, sich gesund zu fühlen, und dem Risiko einer Übertragung von SARS-CoV-2 auf Personen in ihrer unmittelbaren oder weiteren Umgebung.
Befragte Gruppe	Diejenigen, die zum Zeitpunkt der Abgabe der Speichelprobe keine Symptome zeigten, aber dennoch ein positives Testergebnis nach dem PCR-Bestätigungstest hatten
Größe der Stichprobe	Bis maximal 25 Personen
Stichprobenziehung	Zweckmäßige Ziehung aus der quantitativen Studienstichprobe unter den Teilnehmer*innen mit positivem Testergebnis aber ohne Symptomen zum Zeitpunkt der Abgabe der Speichelprobe
Methode	Potentielle Teilnehmer*innen werden rekrutiert, nachdem sie die Testergebnisse erhalten haben. Bei den Methoden wird ein prospektives Format verwendet, das Interviews oder ein anderes qualitatives Element wie z.B. Tagebücher oder Nachrichtendienste einschließt.
Hauptfragestellung	Welche Erfahrungen und Wahrnehmungen haben diejenigen, die positiv auf SARS-CoV-2 getestet wurden aber zum Zeitpunkt des Tests symptomlos waren?
Nebenfragestellungen	• Wie haben Einzelpersonen ihre Diagnose verinnerlicht?<br>• Wie wirkt sich das Erlernen eines asymptomatischen Status auf das Verhalten von Individuen aus?

	• Wie wirkt sich das Erlernen eines asymptomatischen Status auf das Verständnis von SARS-CoV-2/COVID-19 aus?
Rückfragen	Bewältigung, Akzeptanz, Offenheit für zukünftige Tests, Angst, Familie, sozialer Zusammenhalt, Isolation, psychische Gesundheit

Tabelle 2: Zusammenfassung der Interviews für asymptomatische SARS-CoV-2-Träger

Teil 3. Prozess-/Implementierungskomponente

Diese qualitative Teilstudie konzentriert sich auf die Perspektiven derjenigen, die eine Speichelprobe abgeben haben, im Hinblick auf ihre Ansichten über die Testdurchführung und die Pandemie im Allgemeinen.

Perspektiven von Personen, die eine Speichelprobe abgegeben haben	
Übergeordnetes Ziel	Untersuchung der Perspektiven und Erfahrungen derjenigen, die eine Speichelprobe abgegeben haben
Befragte Gruppe	Zur Teilnahme an der Hauptstudie eingeladene Personen, die eine Speichelprobe abgegeben haben
Hauptfragestellung	N=100
Stichprobenziehung	Zufallsstichproben aus den zur Teilnahme an der Hauptstudie eingeladenen Personen, die eine Speichelprobe abgegeben haben
Methode	Kurze, offene Fragen, die in eine Umfrage integriert sind (online und papierbasiert)
Hauptfragestellung	Was sind die Erfahrungen und Wahrnehmungen der Personen die eine Speichelprobe abgegeben haben?
Nebenfragestellungen	• Was hat Sie dazu bewogen, an der Studie mitzumachen/nicht mitzumachen?<br>• Wie fühlte es sich an, eine Speichelprobe abzugeben und in der Studie teilzunehmen?<br>• Wenn Sie bereits eine andere Art von Corona-Test hatten, wie würden Sie diese Erfahrung im Vergleich zu der jetzigen beschreiben (in Bezug auf Probenentnahme, Test, Ergebnisse, Zeit...)?<br>• Wie sehen Sie die allgemeine Testpolitik und den Testansatz in Baden Württemberg?<br>• Was sind Ihre Gedanken zu dieser Pandemie?

Tabelle 3: Zusammenfassung der Umfrage zur Studienteilnahme

14. Rechtliche und ethische Aspekte

14.1. Deklaration von Helsinki

Die Untersuchung wird in Übereinstimmung mit der Deklaration von Helsinki in der aktuellen Fassung durchgeführt. Alle in dieser Studie angewandten Verfahren entsprechen der nationalen und EU-Gesetzgebung in Bezug auf Forschung am Menschen. Die Datenschutzbestimmungen (DSGVO) und des Landesdatenschutzgesetzes Baden-Württemberg (LDSG BW) werden eingehalten.

Nicht einwilligungsfähige Versuchspersonen

An der Studie können auch Kinder ab 7 Jahre und Jugendliche teilnehmen; für beide Personengruppen ist die Einwilligung der Erziehungsberechtigten erforderlich. Die Sars-Cov-2-Pandemie betrifft alle Altersgruppen. Zwar erleiden Kinder und Jugendliche oftmals keine schweren Verläufe von COVID-19, sie sind allerdings insbesondere durch Schulschließungen und Abschottung von Gleichaltrigen bei

Vorliegen einer Infektionslage in der näheren Umgebung betroffen. Falls Infektionen in diesen Altersgruppen frühzeitig entdeckt werden könnten, ließen sich die Isolationsmaßnahmen evtl. auf einzelne Haushalte beschränken und damit die Auswirkungen vor allem auf die physische Gesundheit der Kinder und Jugendlichen minimieren.

Neben der Einwilligung der rechtlichen Vertreter der Kinder und Jugendlichen werden wir auch die Zustimmung der Kinder und Jugendlichen selbst einholen. Eine Ablehnung der Kinder/Jugendlichen ist möglich und wird respektiert.

Körperlich oder geistig zur Einwilligung nicht fähige Personen wie z.B. bewusstlose Menschen/ Patienten werden nicht in die Studie eingeschlossen.

14.2. Ethikkommission

Das Studienprotokoll wird vor Studienbeginn der Ethikkommission der Medizinischen Fakultät Heidelberg zur Begutachtung vorgelegt. Die Studie ist zudem im Deutschen Register für Klinische Studien (DRKS) registriert (UTN U1111-1259-3834). Die Registrierung wird nach Erhalt des Ethikvotums abgeschlossen und das Studienprotokoll publiziert.

14.3. Vertraulichkeit und Datenschutz

Bei allen Arbeiten, die die Erfassung menschlicher Daten, elektronisch oder anderweitig, beinhalten, halten wir uns an die in der Europäischen Richtlinie 95/46/EWG festgelegten Gesetze sowie an die einschlägigen nationalen Gesetze und Vorschriften. Die EU-Richtlinie betrifft den Schutz natürlicher Personen bei der Verarbeitung personenbezogener Daten und den freien Verkehr solcher Daten.

Die Weitergabe von Informationen aus der Studie an Dritte ist auf diejenigen beschränkt, die ein legitimes Peer-Review der medizinischen, wissenschaftlichen und ethischen Aspekte der Studie durchführen, so dass die Zustimmung eingeholt und die übliche medizinische Versorgung gewährleistet werden kann. Die Vertraulichkeit und das Wohlergehen der Studienteilnehmer*innen haben stets oberste Priorität. Alle Personen, die Zugang zu den Daten haben, einschließlich des Prüfarztes/der Prüfärztin, unterliegen während und nach der Studie dem Berufsgeheimnis.

Den Teilnehmern/Teilnehmerinnen wird versichert, dass alle in dieser Studie gewonnenen Informationen vertraulich behandelt werden. Alle Daten (einschließlich demographischer, klinischer und Labordaten) werden in passwortgeschützten Datenbanken gespeichert.

Unser Datenschutzkonzept deckt 2 Phasen der Studie ab, die Datenerhebung und die Datenauswertung:

- <u>Datenerhebung:</u> Die Daten der Teilnehmer*innen (Name, Vorname, Alter, Geschlecht, Adresse) werden von den Einwohnermeldeämtern an das Gesundheitsamt Rhein-Neckar und von dort an den Studienleiter übermittelt. Der Studienleiter führt anhand der Randomisierungsliste von GESIS (Leibniz-Institut für Sozialwissenschaften in Mannheim) die Aufteilung in die vier Wochen-Chargen und in die Studienarme durch und erstellt die notwendigen personalisierten Anschreiben an die Studienteilnehmer*innen. Jedem Studienteilnehmer/jeder -teilnehmerin wird eine eindeutige Identifikationsnummer zugeordnet. Zusätzlich zu den vorhandenen Adress-Daten erhobene Daten werden anhand dieser ID erfasst. In den Armen A1 und A2 werden z.B. freiwillig gemachte Angaben (online, Papier) zu COVID-19-Symptomen sowie Bildungsstatus, Migrationshintergrund und anderen Charakteristika mit dieser ID verknüpft, in den Armen B1 und B2 darüber hinaus die Daten aus dem Pre-Screening Tool. Daten die in der Hotline erhoben werden (Zufriedenheit,

Zeiterfassung, usw.) werden ebenfalls mit dieser ID verknüpft. Bei Vorliegen eines positiven LAMP-Tests wird die Probe pseudonymisiert zur Verifikation an andere Labore (voraussichtlich das Labor der Virologie des Universitätsklinikums) geschickt. Von dort werden positive Fälle entsprechend der Meldepflicht pseudonymisiert an das Gesundheitsamt gemeldet. Der Datenbeauftragte im Gesundheitsamt hat über eine bereits implementierte sicher verschlüsselte Schnittstelle die Möglichkeit, die Klarnamen sowie Telefonnummern und Email-Adressen zu den Pseudonymen aus der Studiendatenbank auszulesen.

- Datenauswertung: Die Adressdaten der Teilnehmer werden nur für die Rekrutierung der Teilnehmer verwendet, nach Vorliegen des Testergebnisses und einer möglichen Kontaktaufnahme für die qualitative Teilstudie werden die Adressdaten, Telefonnummern und Email-Adressen entfernt und die Studiendaten pseudonymisiert (individuell und Haushaltszugehörigkeit); lediglich der Datenbeauftragte der Studie hat über eine separate Masterliste (Pseudonymisierung) Zugriff auf die Klarnamen. Der Zugriff auf die Klarnamen erfolgt nur in medizinisch begründeten Fällen. Für die Datenauswertung werden nur die PLZ, das Alter und das Geschlecht, das Pre-Screening-Ergebnis, das Ergebnis des Labortests sowie operationelle Daten wie z.B. das Datum des Versands der Anschreiben, Datum und Uhrzeit der Probenentnahme und Ankunft der Probe im Labor zugänglich gemacht. Für öffentlich zugängliche Listen werden von der PLZ nur die ersten drei Ziffern bereitgestellt, das Lebensalter von Personen älter als 80 Jahre wird mit „80 oder älter“ kategorisiert.
- Datenerhebung und Auswertung qualitative Teilstudie: Nach Vorliegen des Testergebnisses werden die Studienteilnehmer für die qualitativen Teilstudien rekrutiert. Potentielle Non-Responder werden ab dem 7. Tag seit dem ersten Anschreiben nach Ausbleiben eines Testergebnisses kontaktiert. Die erhobenen Interview- und Survey-Daten werden pseudonymisiert gespeichert und verarbeitet.

Die personenbezogenen Daten unterliegen sowohl der ärztlichen Schweigepflicht als auch den Bestimmungen des Bundesdatenschutzgesetzes (BDSG). Alle Papierfragebögen und Proben werden mit einer Studienidentifikationsnummer versehen und an geeigneten sicheren Orten aufbewahrt. Nur Personen, die das lokal entsprechende Formular zur Datenschutzverpflichtung unterzeichnet haben, erhalten Zugang zu dem passwortgeschützten Computer, auf dem die eingegebenen Daten gespeichert werden. Nach Abschluss des Projekts werden die Daten von den lokalen Computern entfernt und in einer sicheren Datenbank gespeichert. In wissenschaftlichen Publikationen oder Berichten wird kein Patient mit Namen oder Initialen identifiziert. Wenn das Forschungsteam seine Notizen durchsieht, ist es ebenfalls an die berufliche Schweigepflicht gebunden.

Diese Studie schließt Kinder, Frauen und Männer ab 7 Jahren ein. COVID-19 ist eine Krankheit, die Personen aller Altersgruppen betrifft. Nach nationalem Recht ist für Teilnehmer*innen unter dem gesetzlichen Mindestalter ein gesetzlicher Vormund zur Unterzeichnung der Einwilligung erforderlich.

14.4. Freiwilligkeit der Teilnahme

- Die Teilnahme an allen Teilen der Studie ist freiwillig und kostenlose. Die Teilnehmer werden auf die Freiwilligkeit hingewiesen.
- Ein Rücktritt von der Studie und ihren Teilstudien ist jederzeit und aus jedem Grund möglich. Ein Rücktritt von der Studie hat keine Nachteile für eine medizinische Behandlung des Probanden/der Probandin zur Folge.

14.5. Aufklärung/Einwilligung oder Begründung zum Verzicht auf die Einwilligung

Die Studie benötigt sowohl von den Teilnehmer*innen am Pre-Screening als auch von den Probanden/Probandinnen die sich selber eine Speichelprobe entnehmen eine Einwilligung. Die Einwilligung zur Teilnahme am Pre-Screening erfolgt online bzw. papierbasiert; die Einwilligung zur Verwendung der Speichelprobe und der Daten erfolgt papierbasiert. Potenzielle Teilnehmer*innen werden per Post zur Teilnahme an dieser Studie eingeladen. Das Anschreiben erklärt den Zweck der Studie, die potenziell damit verbundenen Risiken und Vorteile der Teilnahme an der Studie und ihre Rechte als Teilnehmer*in (Details zu allen Materialien s. Kapitel 5.2). Die Teilnehmer*innen können ihre schriftliche Einwilligung zusammen mit der Speichelprobe in dem bereitgestellten Rückumschlag ans Studienzentrum schicken.

Um eine Verwendung der Daten für zukünftige Fragestellungen zu ermöglichen, wird in der Einwilligungserklärung eine Formulierung ähnlich der folgenden verwendet: „Die von Ihnen zur Verfügung gestellten oder im Rahmen der Studie erhobenen Daten werden primär für die in dieser Informationsschrift dargelegten Fragestellungen verwendet. In Zukunft können jedoch weitere Untersuchungen mit diesen Daten erforderlich werden, die im Rahmen anderer Forschungsvorhaben behandelt werden. Die genauen Fragestellungen können jedoch zum derzeitigen Zeitpunkt noch nicht konkret benannt werden. Der Forschungszweck wäre jedoch auf folgende Krankheiten/folgende Forschungsgebiete begrenzt: SARS-CoV-2 Infektion, COVID-19. Diese künftigen Forschungsvorhaben werden von der jeweils zuständigen Ethikkommission separat beraten. Eine erneute Aufklärung und Einwilligung Ihrerseits wird nicht erfolgen.“. Die Proben werden nach Vorliegen des Testergebnisses vernichtet.

Qualitative Teilstudie: Die qualitative Teilstudie benötigt sowohl für die Interviews als auch für die Umfrage ebenfalls eine Einwilligung der Studienteilnehmer. Die Einwilligung erfolgt telefonisch nach entsprechendem Hinweis an die Teilnehmer*innen mittels aufgezeichnetem Telefonmitschnitt, elektronisch oder papierbasiert.

14.6. Rücktritt

Ein Proband kann jederzeit ohne Angabe von Gründen seine Teilnahme an der Studie und/oder den Teilstudien abbrechen, ihm/ihr erwächst daraus kein Nachteil.

14.7. Datenvernichtung bei Rücktritt

Bei Vorliegen eines Abbruchwunsches wird der/die Teilnehmer*in befragt, ob die bis zum Zeitpunkt des Abbruchs erhobenen Daten (z.B. Pre-Screening-Daten) verwendet werden dürfen. Falls dies nicht der Fall sein sollte, werden die Daten vernichtet. Die Speichelprobe wird ebenfalls vernichtet und aus der Analyse ausgeschlossen. Falls die Speichelprobe schon analysiert wurde und eine Weiterleitung eines positiven Ergebnisses an das Gesundheitsamt erfolgt ist, wird das Gesundheitsamt über den individuellen Studienabbruch informiert.

14.8. Genehmigung laut RöV/StrSchV

Nicht zutreffend

14.9. Finanzierung, Sponsoren, institutionelle Verbindungen, mögliche Interessenskonflikte, Anreize für Versuchspersonen, Entschädigung

Die Studie ist in das Bundesweite Forschungsnetz "Angewandte Surveillance und Testung" (B-FAST) im Forschungsnetz der Universitätsmedizin zu COVID-19 (NUM) eingebettet und wird direkt vom Bundesministerium für Bildung und Forschung (BMBF) finanziert.

Die direkten Kooperationspartner innerhalb des Forschungsprojektes sind das Heidelberger Institut für Globale Gesundheit, die Sektion Klinische Tropenmedizin des Universitätsklinikums, das Institut für Angewandte Mathematik Heidelberg, das Heidelberg Zentrum für Molekulare Biologie und das Gesundheitsamt Rhein-Neckar. Des Weiteren bestehen innerhalb von B-FAST enge Kooperationen mit dem Institut für Public Health in Mannheim, der Uniklinik Köln, dem Universitätsklinikum Magdeburg, dem Universitätsklinikum Bonn, der Universitätsmedizin Göttingen und dem Universitätsklinikum Schleswig-Holstein in Lübeck.

Anreize für Versuchspersonen/Entschädigungen

Anreize und Entschädigungen für Studienteilnehmer sind nicht geplant. Durch Anreize könnte evtl. die Teilnahmebereitschaft erhöht werden. Da wir allerdings eine Strategie identifizieren wollen, die sich nachher für den Routine-Einsatz eignet, verzichten wir auf Anreize, um die Studienergebnisse gegenüber einem potentiellen Routine-Betrieb nicht zu verzerren.

15. Literaturverzeichnis

[1] Chan JFW, Yuan S, Kok KH, To KKW, Chu H, Yang J, et al. A familial cluster of pneumonia associated with the 2019 novel coronavirus indicating person-to-person transmission: a study of a family cluster. The Lancet. 2020;395(10223):514–523.

[2] Wang Y, Liu Y, Liu L, Wang X, Luo N, Ling L. Clinical outcome of 55 asymptomatic cases at the time of hospital admission infected with SARS-Coronavirus-2 in Shenzhen, China. The Journal of infectious diseases. 2020;.

[3] JHU. Coronavirus COVID-19 Global Cases by the Center for Systems Science and Engineering (CSSE) at Johns Hopkins University; 2020. Zugriff 30.09.2020; https://gisanddata.maps.arcgis.com/apps/opsdashboard/index.html#/bda7594740fd40299423467b48e9ecf6.

[4] WHO. WHO Director-General’s opening remarks at the media briefing on COVID-19 -11 March 2020; 2020. Zugriff 30.09.2020; https://www.who.int/dg/speeches/detail/who-director-general-s-opening-remarks-at-the-media-briefing-on-covid-19—11-march-2020.

[5] Paules CI, Marston HD, Fauci AS. Coronavirus infections - more than just the common cold. Jama. 2020;323(8):707–708.

[6] Chen J. Pathogenicity and transmissibility of 2019-nCo: a quick overview and comparison with other emerging viruses. Microbes and infection. 2020;.

[7] Zhou P, Yang XL, Wang XG, Hu B, Zhang L, Zhang W, et al. A pneumonia outbreak associated with a new coronavirus of probable bat origin. nature. 2020;579(7798):270–273.

[8] Zou L, Ruan F, Huang M, Liang L, Huang H, Hong Z, et al. SARS-CoV-2 viral load in upper respiratory specimens of infected patients. New England Journal of Medicine. 2020;382(12):1177–1179.

[9] Tabata S, Imai K, Kawano S, Ikeda M, Kodama T, Miyoshi K, et al. Clinical characteristics of COVID-19 in 104 people with SARS-CoV-2 infection on the Diamond Princess cruise ship: a retrospective analysis. The Lancet Infectious Diseases. 2020;.

[10] Corman VM, Landt O, Kaiser M, Molenkamp R, Meijer A, Chu DK, et al. Detection of 2019 novel coronavirus (2019-nCoV) by real-time RT-PCR. Eurosurveillance. 2020;25(3):2000045.

[11] Deckert A, Bärnighausen T, Kyei NN. Simulation of pooled-sample analysis strategies for COVID-19 mass testing. World Health Organization Bulletin of the World Health Organization. 2020;98(9):590–598.

[12] Kojima N, Turner F, Slepnev V, Bacelar A, Deming L, Kodeboyina S, et al. Self-Collected Oral Fluid and Nasal Swabs Demonstrate Comparable Sensitivity to Clinician Collected Nasopharyngeal Swabs for Covid-19 Detection. medRxiv. 2020;.

[13] Wyllie AL, Fournier J, Casanovas-Massana A, Campbell M, Tokuyama M, Vijayakumar P, et al. Saliva is more sensitive for SARS-CoV-2 detection in COVID-19 patients than nasopharyngeal swabs. Medrxiv. 2020;.

[14] Goldfarb DM, Tilley P, Al-Rawahi GN, Srigley J, Ford G, Pedersen H, et al. Self-collected Saline Gargle Samples as an Alternative to Healthcare Worker Collected Nasopharyngeal Swabs for COVID-19 Diagnosis in Outpatients. medRxiv. 2020;.

[15] Malecki M, Lüsebrink J, Teves S, Wendel AF. Pharynx gargle samples are suitable for SARS-CoV-2 diagnostic and save personal protective equipment and swabs. Infection Control & Hospital Epidemiology. 2020;p. 1–7.

[16] Robert Koch Institut. Hinweise zur Testung von Patienten auf Infektion mit dem neuartigen Coronavirus SARS-CoV-2; 2020. Zugriff 05.10.2020; https://www.rki.de/DE/Content/InfAZ/N/Neuartiges_Coronavirus/Vorl_Testung_nCoV.html.

[17] Thi VLD, Herbst K, Boerner K, Meurer M, Kremer LP, Kirrmaier D, et al. Screening for SARS-CoV-2 infections with colorimetric RT-LAMP and LAMP sequencing. medRxiv. 2020;.

[18] Kellner MJ, Ross JJ, Schnabl J, Dekens MP, Heinen R, Grishkovskaya I, et al. A rapid, highly sensitive and open-access SARS-CoV-2 detection assay for laboratory and home testing. 2020;.

[19] Zhang Y, Ren G, Buss J, Barry AJ, Patton GC, Tanner NA. Enhancing Colorimetric LAMP Amplification Speed and Sensitivity with Guanidine Chloride. bioRxiv. 2020;.

[20] Ott IM, Strine MS, Watkins AE, Boot M, Kalinich CC, Harden CA, et al. Simply saliva: stability of SARS-CoV-2 detection negates the need for expensive collection devices. medRxiv. 2020;.

[21] Cox LH. A constructive procedure for unbiased controlled rounding. Journal of the American Statistical Association. 1987;82(398):520–524.

[22] Koeppel L, Gottschalk C, Welker A, Knorr B, Denkinger CM. Prediction of local COVID-19 spread in Heidelberg. F1000Research. 2020;9(232):232.

[23] Brown LD, Cai TT, DasGupta A. Interval estimation for a binomial proportion. Statistical science. 2001;p. 101–117.

[24] Bundesinstitut für Bevölkerungsforschung. Zahl der Privathaushalte und durchschnittliche Haushaltsgröße in Deutschland (1991-2035); 2020. Zugriff 04.10.2020;

https://www.bib.bund.de/DE/Fakten/Fakt/L50-Privathaushalte-Haushaltsgroesse-1991-Vorausberechnung.html.

[25] Häder S, et al. Stichproben in der Praxis. Mannheim, GESIS–Leibniz-Institut für Sozialwissenschaften (GESIS Survey Guidelines). 2015;10.

[26] Zhang Z. Introduction to machine learning: k-nearest neighbors. Annals of translational medicine. 2016;4(11).

[27] Sussman JB, Hayward RA. An IV for the RCT: using instrumental variables to adjust for treatment contamination in randomised controlled trials. Bmj. 2010;340:c2073.

[28] Zou G. A modified poisson regression approach to prospective studies with binary data. American journal of epidemiology. 2004;159(7):702–706.
